# Entanglement monogamy in indistinguishable particle systems

**DOI:** 10.1038/s41598-023-46515-z

**Published:** 2023-12-11

**Authors:** Soumya Das, Goutam Paul, Ritabrata Sengupta

**Affiliations:** 1 Cryptology and Security Research Unit, R. C. Bose Centre for Cryptology and Security, Indian Statistical Institute, Kolkata, 700108 India; 2 Department of Mathematical Sciences, Indian Institute of Science Education and Research Berhampur, Transit Campus, Government ITI, Berhampur, Odisha 760010 India

**Keywords:** Physics, Quantum physics, Quantum information

## Abstract

Recently, it has been realized that indistinguishability is a resource for quantum information processing. A new method to represent the indistinguishable particles by Franco et al. (Sci Rep 6:20603, 2016, 10.1038/srep20603) and measure the concurrence is developed by Nosrati et al. (npj Quantum Inf 6:39, 2020, 10.1038/s41534-020-0271-7). The monogamy property says that quantum entanglement cannot be shared freely between more than two particles. For three distinguishable particles, the monogamy of entanglement was first expressed as an inequality using squared concurrence where each particle has a single degree of freedom (for pure or mixed states). Using multiple degrees of freedom, similar inequality was shown to be held between two distinguishable particles. However, for two indistinguishable particles, where each particle cannot be addressed individually, the monogamy inequality was shown to be violated maximally for a specific state. Thus a question naturally arises: what happens to the monogamy of entanglement in the case of three or more indistinguishable particles? We prove that monogamy holds in this scenario and the inequality becomes equality for all pure indistinguishable states. Further, we provide three major operational meanings of our result. Finally, we present an experimental schematic using photons to observe our result.

## Introduction

Quantum entanglement is a fundamental concept in quantum information that is used in many quantum protocols. Quantum information is generally encoded in a particle’s degree of freedom (DoF) like spin, orbital angular momentum (OAM) etc.^1^, and entanglement usually deals with particles having a single DoF^2,3^. A few recent works have considered multiple DoFs of a single particle to study what is called inter-DoF entanglement^4–13^, albeit in the context of distinguishable particles. For indistinguishable particles^14–28^, where each particle cannot be addressed individually^29,30^, (i.e., a label cannot be associated with each particle) the characterization of inter-DoF entanglement requires a different analysis^31–38^.

An interesting feature of entanglement is its restriction upon the shareability among several particles, known as the *monogamy of entanglement* (MoE), first expressed in Ref.^39^ using squared concurrence (${\mathscr{C}}^2$)^40^ as the entanglement measure. The monogamy inequality with respect to *A* for a three-particle state $\rho _{ABC}$ can be written as

1 $$\begin{aligned} {\mathscr {C}}^{2}_{A|B} \left( \rho _{AB} \right) + {\mathscr {C}}^{2}_{A|C}\left( \rho _{AC}\right) \le {\mathscr {C}}^{2}_{A|BC} \left( \rho _{ABC} \right) , \end{aligned}$$

where $\rho _{AB}=\text {Tr}_{C} \left( \rho _{ABC}\right)$, $\rho _{AC}=\text {Tr}_{B} \left( \rho _{ABC}\right)$, and ${\mathscr {C}}_{X|Y}$ measures the concurrence between systems *X* and *Y* of the composite system *XY*, where the vertical bar represents bipartite splitting.

Equation (1) considers entanglement involving a single DoF of each of three particles and views a particle and its associated DoF as the same entity. We call this type of MoE as *particle-MoE* and it can be generalized to inter-DoF MoE^37^ (in short, DoF-MoE) as follows. Consider three entities *A*, *B*, and *C*, each with *n* DoFs, numbered 1 to *n*. If the joint state of the *i*th, *j*th, and *k*th DoFs of *A*, *B*, and *C* respectively is represented by $\rho _{A_{i}B_{j}C_{k}}$, then the DoF-MoE with respect to the *i*th DoF of *A* is stated as follows.

2 $$\begin{aligned} {\mathscr {C}}^{2}_{A_{i}|B_{j}}(\rho _{A_{i}B_{j}})+{\mathscr {C}}^{2}_{A_{i}|C_{k}}(\rho _{A_{i}C_{k}}) \le {\mathscr {C}}^{2}_{A_{i}|B_{j}C_{k}}(\rho _{A_{i}B_{j}C_{k}}), \end{aligned}$$

where $\rho _{A_{i}B_{j}}=\text {Tr}_{C_{k}} ( \rho _{A_{i}B_{j}C_{k}})$, $\rho _{A_{i}C_{k}}=\text {Tr}_{B_{j}} ( \rho _{A_{i}B_{j}C_{k}})$. This generalized representation covers multiple scenarios such as (i) three particles (this case coincides with particle-MoE in Eq. (1)), (ii) two particles (when *B* and one of *A*/*C* becomes the same particle), as well as (iii) one particle (when *A*, *B*, and *C* denote the same particle) as shown in Ref.^37^. One may think that different DoFs are equivalent to different particles, but this is not true in general (see Supplemental Information 1 for more details).

There is a fundamental difference between the physicality of the entanglement of distinguishable particles and that of indistinguishable ones. For example, two distinguishable particles with orthogonal eigenstates in one of the DoFs are separable as they can be written in the tensor product. However, the same for two indistinguishable particles become entangled Methods in Ref.^31^, which is also experimentally verified in Ref.^41^ (see Supplemental Information 2 for more details). So, if three or more particles become indistinguishable in the same/different localized regions in their same/different eigenstates of same/different DoFs in an arbitrary manner, whether MoE holds or not is not immediately obvious and needs non-trivial analysis. This is the motivation behind this article.

For distinguishable particles, MoE is known to hold, irrespective of whether the DoFs involved come from two particles^11,12,37^ or more^39,42^. For two indistinguishable particles, it has been shown that monogamy does not necessarily hold and can be violated maximally^37^. So a natural question arises, whether MoE always holds for three or more indistinguishable particles or not?

In this article, we show that monogamy of entanglement holds for three or more indistinguishable particles each having single or multiple DoFs using squared concurrence as the entanglement measure. The validity of monogamy under different scenarios is depicted in Table 1. Specifically, we show that for pure indistinguishable states, the monogamy inequality becomes equality, whereas inequality remains for mixed states. We present other three major operational meanings for our result, Firstly, a strict monogamy inequality for pure states implies that the particles are distinguishable. Secondly, a strict monogamy inequality for indistinguishable particles implies that the particles are in a mixed state. Finally, If monogamy equality does not hold for any unknown quantum state, then the state cannot be both pure and made of indistinguishable particles. To verify our proposal experimentally, we present an optical schematic using photons to demonstrate our result.

**Table 1:** Summary of the results related to monogamy of entanglement for distinguishable and indistinguishable particles.

	Distinguishable	Indistinguishable
2 particles	Holds^11,12^	Can violate maximally^37^
$\ge$ 3 particles	Holds^39,42^	Holds (This Article)

## Results

### Representation of the general state of *p* indistinguishable particles each having *n* DoFs

Here, we revisit the formulation of Refs.^31,32,37^ in a more general setting, with explicit consideration of the Pauli exclusion principle^43^.

We describe the general state of *p* indistinguishable particles each having *n* degrees of freedom. The *P* spatial labels are represented by $\alpha ^{i}$ that ranges over ${\mathbb {S}}^P:= \lbrace s^1, s^2, \ldots , s^P \rbrace$. We write the set $\lbrace 1, 2, \ldots , n \rbrace$ as ${\mathbb {N}}_n$. Here $a^{i}_{j}$ ranges over ${\mathbb {D}}_{j}:=\lbrace D_{j_{1}}, D_{j_{2}}, \ldots , D_{j_{k_{j}}} \rbrace$, represents the eigenvalue of the *j*-th DoF of the particle in the $\alpha ^{i}$-th localized region where $j \in {\mathbb {N}}_n$. Thus the general state of *p* indistinguishable particles each having *n* DoFs is defined as

3 $$\begin{aligned} {|{\Psi ^{\left( p,n\right) }}\rangle }:= \sum _{\alpha ^{i}, a^{i}_{j} } \eta ^u \kappa ^{\alpha ^{1}, \alpha ^{2}, \ldots , \alpha ^{p}}_{a^{1}_{1}a^{1}_{2} \ldots a^{1}_{n}, a^{2}_{1}a^{2}_{2} \ldots a^{2}_{n},\ldots , a^{p}_{1}a^{p}_{2} \ldots a^{p}_{n}} {|{\alpha ^{1} a^{1}_{1}a^{1}_{2} \ldots a^{1}_{n}, \alpha ^{2} {a^{2}_{1}a^{2}_{2} \ldots a^{2}_{n}, \ldots , \alpha ^{p} a^{p}_{1} a^{p}_{2} \ldots a^{p}_{n} }}\rangle }. \end{aligned}$$

Here *u* represents the summation of parity of the cyclic permutations of all the *n* DoFs. Thus *u* can be represented as $u=u_1+u_2+ \cdots + u_n=\sum _i u_j$ where $u_j$ is the parity of the *j*-th DoF. The value of $\eta$ is $+1$ for bosons and $-1$ for fermions. If we have the following condition that

$$\begin{aligned} \left( \alpha ^{i}=\alpha ^{i^\prime } \right) \wedge \left( a^{i}_{j}=a^{i^{\prime }}_{j} \right) , \end{aligned}$$

for any $i\ne i^{\prime }$ where $\alpha ^i , \alpha ^{i^{\prime }} \in {\mathbb {S}}^{P}$ and $j \in {\mathbb {N}}_{n}$, then we get $\eta =0$ for fermions due to Pauli exclusion principle^43^.

Following the above notations, the general density matrix of *p* indistinguishable particles each having *n* DoFs is defined as

4 $$\begin{aligned} \rho ^{(p, n)}:= \sum _{\begin{array}{c} \alpha ^{i}, \beta ^{i}, a^{i}_{j}, b^{i}_{j} \end{array}} \eta ^{(u+{\bar{u}})} \kappa ^{ \alpha ^{(p)}}_{a_{(n)} } \kappa ^{ \beta ^{(p)}* }_{ b_{(n)} } {|{\alpha ^{1} a^{1}_{1}a^{1}_{2} \ldots a^{1}_{n}, \alpha ^{2} {a^{2}_{1}a^{2}_{2} \ldots a^{2}_{n}, \ldots , \alpha ^{p} a^{p}_{1} a^{p}_{2} \ldots a^{p}_{n} }}\rangle } {\langle {\beta ^{1} b^{1}_{1}b^{1}_{2} \ldots b^{1}_{n}, \beta ^{2} {b^{2}_{1}b^{2}_{2} \ldots b^{2}_{n}, \ldots , \beta ^{p} b^{p}_{1} b^{p}_{2} \ldots b^{p}_{n} }}|}, \end{aligned}$$

where

$$\begin{aligned}  \kappa ^{\alpha ^{(p)}}_{a_{(n)} }&=\kappa ^{\alpha ^{1}, \alpha ^{2}, \ldots , \alpha ^{p}}_{a^{1}_{1}a^{1}_{2} \ldots a^{1}_{n}, a^{2}_{1}a^{2}_{2} \ldots a^{2}_{n}, \ldots , a^{p}_{1}a^{p}_{2} \ldots a^{p}_{n}}, \hspace{1cm} \kappa ^{\beta ^{(p)}}_{b_{(n)} }=\kappa ^{\beta ^{1}, \beta ^{2}, \ldots , \beta ^{p}}_{b^{1}_{1}b^{1}_{2} \ldots b^{1}_{n}, b^{2}_{1}b^{2}_{2} \ldots b^{2}_{n}, \ldots , b^{p}_{1}b^{p}_{2} \ldots b^{p}_{n}}, \end{aligned}$$

and $\alpha ^{i}, \beta ^{i}$ ranges over ${\mathbb {S}}^p$, $a^{i}_{j}, b^{i}_{j}$ ranges over ${\mathbb {D}}_{j}$, $i \in {\mathbb {N}}_P$ and $j \in {\mathbb {N}}_n$. Here *u* is as defined in Eq. (3) and ${\bar{u}}$ comes due to the density matrix. If we have the following condition that

$$\begin{aligned} \left\{ \left( \alpha ^{i}=\alpha ^{i^\prime } \right) \vee \left( a^{i}_{j}=a^{i^{\prime }}_{j} \right) \right\} \wedge \left\{ \left( \beta ^{i}=\beta ^{i^\prime } \right) \vee \left( b^{i}_{j}=b^{i^{\prime }}_{j} \right) \right\} , \end{aligned}$$

for any $i \ne i^{\prime }$ where $i, i^{\prime } \in {\mathbb {N}}_P$ and $j \in {\mathbb {N}}_{n}$, then we get $\eta =0$ for fermions due to Pauli exclusion principle^43^.

### Monogamy of entanglement for indistinguishable particles

In this section, we present our main result. As the state-space structure of distinguishable and indistinguishable particles are completely different, the proof for MoE for distinguishable particles^39^ is not applicable to indistinguishable ones. So, we calculate the MoE for all the possible ways in which indistinguishability can occur.

For the sake of brevity and ease of understanding, here in “Proof of MoE for three indistinguishable particles each having a single DoF” section, we prove MoE for three indistinguishable particles each having a single DoF with two eigenvalues. For example, take the spin DoF with eigenstates $\lbrace {|{\uparrow }\rangle }, {|{\downarrow }\rangle } \rbrace$ in three localized regions ${\mathbb {S}}^3= \lbrace s^1, s^2, s^3 \rbrace$.

Next, we repeat the above calculations of MoE by increasing the number of DoFs from one to two in “Proof of MoE for three indistinguishable particles each having two DoFs” section. For example, take the DoFs as spin and OAM with eigenstates $\lbrace {|{\uparrow }\rangle }, {|{\downarrow }\rangle } \rbrace$ and $\lbrace {|{+l}\rangle }, {|{-l}\rangle } \rbrace$ respectively. Analysis of this situation results in five major cases where one of the eigenstates of the DoFs contributes for entanglement, and the other non-contributing DoFs take arbitrary values. Then we consider the other cases where contributing DoFs for entanglement can be in an arbitrary superposition of their eigenstates.

Finally, we perform the calculation of MoE for the most general situation by taking an arbitrary number of particles and each having an arbitrary number of DoFs in “[Sec Sec30]” section. We take *p* $(\ge 3)$ indistinguishable particles each having *n* DoFs. This situation yields thirteen non-trivial cases.

In all the above situations, monogamy holds for pure states with an equality relation. We encourage the reader to go through the first situation in “Proof of MoE for three indistinguishable particles each having a single DoF” section, then the second in “Proof of MoE for three indistinguishable particles each having two DoFs” section, and finally the general situation in in “[Sec Sec30]” section.

On the other hand, for mixed states, we use the convexity of concurrence to prove the monogamy inequality in “Proof of MoE indistinguishable particles for mixed states” section. Expressing any mixed state as an ensemble of the pure states, we apply the concurrence on each such pure state and do a minimization to get the required inequality for any arbitrary mixed states.

Thus the following result holds for all pure and mixed indistinguishable particles.

#### Result 1

Three or more indistinguishable particles, each having an arbitrary number of degrees of freedom, obey the monogamy of entanglement using squared concurrence.

Although MoE holds for both distinguishable and indistinguishable particles, the derivation of our result reveals a fundamental difference between them as stated below.

#### Corollary 1.1

If monogamy is calculated using three (or more) indistinguishable particles, then for all pure states we can write Eq. (2) as

5 $$\begin{aligned} {\mathscr {C}}^{2}_{\alpha _{i}|\beta _{j}}(\rho _{\alpha _{i}\beta _{j}})+{\mathscr {C}}^{2}_{\alpha _{i}|\gamma _{k}}(\rho _{\alpha _{i}\gamma _{k}}) = {\mathscr {C}}^{2}_{\alpha _{i}|\beta _{j}\gamma _{k}}(\rho _{\alpha _{i}\beta _{j}\gamma _{k}}), \end{aligned}$$

where $\alpha , \beta , \gamma$ are spatial locations and *i*, *j*, *k* denote the DoF indices $\in {\mathbb {N}}_n$ following the notations in Eq. (3). Corollary 5 can be extended to more than three particles as shown in Result 1. The physical significance of this result is that for all pure states, if MoE is calculated using squared concurrence for three or more indistinguishable particles, then the residual entanglement in the whole state is zero.

The broad picture given by our result is summarized in Table 2. It can be seen that monogamy equality holds only for pure indistinguishable particles. For the other cases, monogamy inequality holds.

**Table 2:** Operational meaning of our result.

	Distinguishable	Indistinguishable
Pure	Inequality ($\le$) Holds	Equality ($=$) Holds
Mixed	Inequality ($\le$) Holds	Inequality ($\le$) Holds

Here we see that MoE equality holds for only pure indistinguishable particles using three or more particles and taking concurrence as an entanglement measure. For the rest of the cases, the MoE inequality holds.

Thus we give a clear distinction of some property that is possible using distinguishable particles and prove that is impossible using indistinguishable particles. In Ref.^39^, it was proved that a strict monogamy inequality is possible using pure distinguishable particles. Our result proves that a strict inequality is not possible using pure indistinguishable particles.

## Operational meaning of our result

Suppose we have an unknown density matrix $\rho$ consisting of three or more particles. Now the question is how Corollary 5 is operationally useful to characterize this density matrix $\rho$ based on the purity and distinguishability? We will perform the monogamy equality test, i.e., whether Corollary 5 is satisfied or not as described below to find the answer.

*Case 1* Suppose we have a state that is both pure and indistinguishable. Then according to Corollary 5, that state will follow monogamy equality.

*Case 2* Suppose we have an unknown pure state ${|{\psi }\rangle }$ where ${|{\psi }\rangle }{\langle {\psi }|}=\rho$ and no information is given about its distinguishability. Now if we perform the monogamy equality test and we get that $\rho$ holds a strictly less than relation (<), i.e., Corollary 5 is not satisfied, then $\rho$ is a distinguishable state.

*Case 3* Suppose we have an unknown indistinguishable density matrix $\rho$ where no information is given about its purity. Now if we perform the monogamy equality test and we get that $\rho$ holds a strictly less than relation (<), i.e., Corollary 5 is not satisfied, then $\rho$ is not a mixed state.

*Case 4* Suppose we have an unknown density matrix $\rho$ where no information is given about its purity and distinguishability. Now if we perform the monogamy equality test and we get that $\rho$ holds a strictly less than relation (<), i.e., Corollary 5 is not satisfied, then $\rho$ cannot both pure and made of indistinguishable particles.

**Figure 1:**
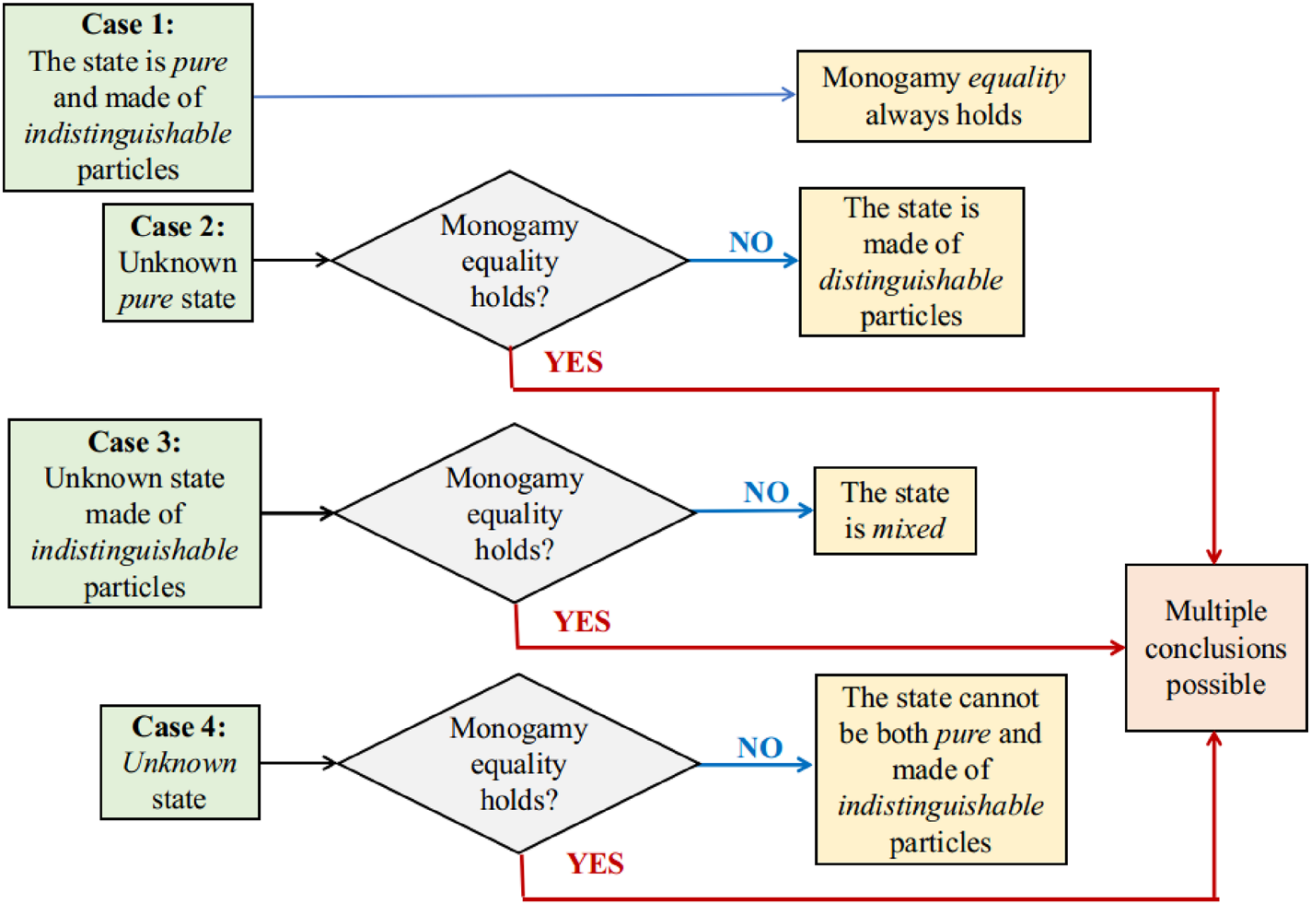
Operational meaning of Corollary 5 having four implications. (1) Any pure and indistinguishable quantum state obeys monogamy equality. (2) If monogamy equality does not hold for any pure quantum state, then the state is made of distinguishable particles. (3) If monogamy equality does not hold for a quantum state made of indistinguishable particles, then the state is a mixed state. (4) If monogamy equality does not hold for any unknown quantum state, then the state cannot be both pure and made of indistinguishable particles.

The significance of our result is that it establishes a connection between the three properties, say *monogamy*, *purity*, and *distinguishability* of a specific type of density matrix. A flowchart of all these cases is shown in Fig. 1.

One may argue that purity can be checked easily using the SWAP test and randomized measurements^44^. So, why do we need to perform the tests mentioned in Fig. 1? The answer is that the SWAP tests are possible for distinguishable particles only, as it requires controlled *NOT* gates. However, for indistinguishable cases, as each particle cannot be addressed individually, we cannot perform the SWAP test. It must be noted that in certain Bose–Einstein condensation scenarios, parity checking was performed as in Ref.^45^. Whether such tests can be performed in all indistinguishable cases is not worked out as per our knowledge. For randomized measurements, ideally, an infinite number of copies are needed. But for the test mentioned in this paper, ideally, one single copy is needed. It must also be noted that there is no known method to check whether the particles are distinguishable or not, for any arbitrary unknown state.

## An experimental scheme to observe monogamy equality for pure states using three indistinguishable photons

In Result 1, we have theoretically proved that three or more indistinguishable particles always obey a monogamy equality relation. Here, we present an experimental schematic using three indistinguishable photons to illustrate our result. One can also create more circuits to illustrate our result experimentally.

**Figure 2:**
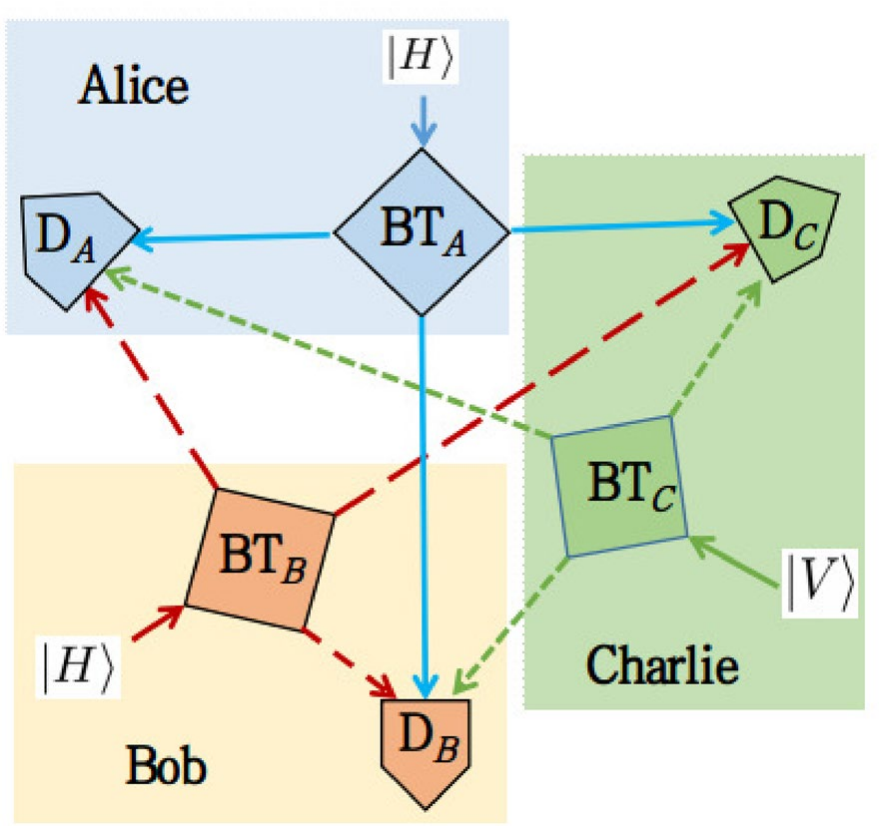
We present an experimental schematic using three indistinguishable photons to illustrate the equality monogamy relation. This state is analogous to the W-type state of distinguishable particles. Here, three parties Alice, Bob, and Charlie send three photons with ${|{H}\rangle }$, ${|{H}\rangle }$, and ${|{V}\rangle }$ eigenstate respectively in polarization DoF to three beam tritters (BT) denoted by $\hbox {BT}_{A}$, $\hbox {BT}_B$, and $\hbox {BT}_C$ respectively. From each beam tritter, the photons are received in the detectors denoted by $\hbox {D}_A$, $\hbox {D}_B$, and $\hbox {D}_C$ which belong to Alice, Bob, and Charlie respectively. The detection procedure of the photons is the same as for distinguishable ones (see Supplemental Information 3). The only difference is that we do not know which photons are being detected.

For simplicity, we present this scheme using only the polarization DoF of the photon with eigenstates $\lbrace {|{H}\rangle }, {|{V}\rangle } \rbrace$. This can be extended to *p* number of indistinguishable photons having *n* number of DoFs. Assume Alice and Bob have two photons in ${|{H}\rangle }$ eigenstate and Charlie has a photon in ${|{V}\rangle }$ eigenstate. The three photons go to the respective beam tritters (BT) denoted by $\hbox {BT}_{A}$, $\hbox {BT}_B$, and $\hbox {BT}_C$ respectively whose three output ports go to all of the three detectors $D_A$, $D_B$, and $D_C$, as shown in Fig. 2. This is essentially a particle exchange method^13,31^ to produce indistinguishable particles. Here, we will consider only those cases where each of the detectors detects only one photon. Note that, the measurements for indistinguishable particles are the same as for distinguishable ones

Here, the beam tritter is a generalization of the beam splitter for higher dimensions. The theoretical modeling of the beam tritters can be found in Ref.^46,47^ with applications^48,49^. Experimental realization of the beam tritters can be found in Ref.^50,51^. The transition matrix for each of the beam tritters can be written as^46^

$$\begin{aligned} \frac{1}{\sqrt{3}} \begin{pmatrix} 1 &{} 1 &{} 1\\ 1 &{} \omega &{} \omega ^{2} \\ 1 &{} \omega ^{2} &{} \omega ^{4} \end{pmatrix}, \end{aligned}$$

where $$\omega = exp(\frac{i 2 \pi }{3})$$.

Let three localized regions $s^{1}$, $s^{2}$, and $s^{3}$ belongs to Alice, Bob and Charlie where the detectors $D_A$, $D_B$, and $D_C$ are present. The initial state of the particles can be written as ${|{\Psi ^{(3,1)}}\rangle }_{i}={|{H}\rangle }_{A}\otimes {|{H}\rangle }_{B}\otimes {|{V}\rangle }_{C}$. After particle exchange, the final state can be written using the notations of Eq. (26) as

6 $$\begin{aligned}  {|{\Psi ^{(3,1)}}\rangle }_{f}= \frac{1}{\sqrt{3}}&\big ( {|{s^1 H, s^2 H, s^3 V }\rangle } + \eta {|{s^1 H, s^2 V, s^3 H }\rangle } + {|{s^1 V, s^2 H, s^3 H }\rangle } \big ). \end{aligned}$$

Now we can calculate the monogamy following the calculations in the “Proof of MoE for three indistinguishable particles each having a single DoF” section. After calculation we get ${\mathscr {C}}^{2}_{s^1 \mid s^2} + {\mathscr {C}}^{2}_{s^1 \mid s^3}={\mathscr {C}}^{2}_{s^1 \mid s^2 s^3} = \frac{8}{9}$.

One may think that whether it will be possible to create states that follow a strict monogamy inequality relation using indistinguishable particles. The answer is no. In Supplemental Information 4, we show the condition for a general three-qubit state using distinguishable particles that follow a strict monogamy inequality relation and why those states cannot be generated using indistinguishable particles.

Note that, the state we have created in Eq. (6) is analogous to the W-type state of distinguishable particles^52^. However, none of the existing literature has shown how to create this type of state using indistinguishable particles. This is the first contribution of this setup. Secondly, this W-type of state gives a strict monogamy equality relation for distinguishable particles. We have shown for indistinguishable particles, the results exactly the same as shown in the first row of Table 2.

## Discussion

Quantum mechanics features the existence of particles that are indistinguishable, which has drawn significant attention within the scientific community. These indistinguishable particles are being explored as a resource^28^ for various quantum information processing tasks, including teleportation^32,53^ and entanglement swapping^54^, which are traditionally carried out using distinguishable particles.

A recent series of published findings have highlighted the unique properties and applications that are specific to indistinguishable or distinguishable particles, referred to as “separation results” between these two categories. Das et al.^38^ demonstrated that only distinguishable particles can achieve unit fidelity quantum teleportation, while only indistinguishable particles can produce hyper-hybrid entangled states. In cases where a quantum protocol can be executed using both types of particles, one may offer advantages over the other. For instance, entanglement swapping requires a minimum of two indistinguishable particles^38^, whereas three distinguishable particles are needed^55,56^. Another separation result by Paul et al.^37^. reveals that using two indistinguishable particles, each with multiple degrees of freedom, can maximally violate monogamy of entanglement, which is not feasible with distinguishable particles^11^.

Building upon the aforementioned separation results, this article presents a distinct property of indistinguishable particles that sets them apart from distinguishable ones. Specifically, the inequality of the MoE using squared concurrence for three or more distinguishable particles, as depicted in Ref.^39^, becomes an equality for pure indistinguishable states. However, this equality may only hold for mixed indistinguishable states. It is worth noting that this equality differs from the one proposed in Ref.^57^. This finding proves particularly useful in calculating entanglement in scenarios where particles are indistinguishable, such as in quantum dots^58,59^, ultracold atomic gases^60^, Bose–Einstein condensates^61,62^, quantum meteorology^63,64^, among others.

The significance of our result is that it establishes a connection between the three properties, say monogamy, purity, and distinguishability of some specific quantum states. For example, if an unknown pure state obeys strict monogamy inequality implies that the state is made of distinguishable particles. Also, if an unknown state made of indistinguishable particles obeys a strict monogamy inequality implies that the particles are in a mixed state. The full characterization of all the states based on monogamy, purity, and distinguishability is an interesting future work.

## Methods

## Revisiting the representation and definition of entanglement for indistinguishable particles, DoF trace-out rule and calculation of concurrence for indistinguishable particles

Here, we revisit the representation and definition of entanglement for indistinguishable particles^31,32^, the existing results of DoF trace-out for indistinguishable particles^37,65^ and the calculation of the concurrence between any two DoFs of two indistinguishable particles^66^ with the representation described in “Representation of the general state of *p* indistinguishable particles each having *n* DoFs” section.

### The representation and definition of entanglement for indistinguishable particles

The central challenge in the field of quantum information theory lies in the inadequacy of conventional entanglement measures when applied to identical particle states^14–23,25,27,67^. Traditionally, metrics such as the von Neumann entropy of the reduced state are unable to distinguish between entanglement and the mere independence of separated particles. This issue creates conflicting outcomes for bosons and fermions^68–78^. It’s worth noting that this challenge is not exclusive to the particle-based (first quantization) description^14–19,21,23^ but also applies to the mode-based (second quantization) approach^20,22,22,67^, where name labels are not explicitly mentioned but are implicitly assumed.

This problem has driven the development of alternative methods for identifying entanglement among identical particles^16,18,21,24,79–83^. These methods depart from the conventional ones used for nonidentical particles, either by redefining the concept of entanglement or by seeking tensor product structures supported by observables. The goal is to distinguish the physically relevant entanglement from the unphysical components. The need for such novel approaches to address quantum correlations for identical and nonidentical particles is somewhat surprising. However, these approaches remain somewhat cumbersome from a technical standpoint and are less suitable for quantifying entanglement under general conditions of scalability or in realistic scenarios where identical particles are in close proximity, leading to spatial overlap.

In quantum mechanics, identical particles are assigned name-labels to make them distinguishable. To ensure that this fictitious system behaves like a real bosonic or fermionic system, only symmetrized or antisymmetrized states with respect to the labels are permitted^84,85^. While this approach generally works well in practice, complications arise when dealing with entanglement, which critically depends on the form of the state vector. This complexity arises from the simultaneous contributions of real and fictitious (label-born) factors to the entangled state.

In our work, we have taken a recent approach^31,32,54^ that aims to provide a more straightforward description of quantum correlations in identical particle systems, grounded in simple physical principles that can unequivocally address the fundamental question: when and to what extent does the indistinguishability of quantum particles become physically relevant in determining their entanglement? They represent an approach to identical particles that, like second quantization, dispenses with name labels while adopting a particle-based (first quantization) formalism based on states. This approach treats a many-particle state as a single entity characterized by a complete set of commuting observables. It quantifies the physical entanglement of both bosons and fermions using the same principles employed for distinguishable particles, such as the von Neumann entropy of the partial trace. This approach enables the study of identical particle entanglement under arbitrary conditions of wave function overlap at the same level of complexity required for nonidentical particles. Furthermore, by imposing the condition of spatially separated (i.e., non-overlapping) particles, our approach recovers known results for distinguishable particles.

If the state vector of two indistinguishable particles is labeled by $\phi$ and $\psi$, then the two-particle state is represented by a single entity ${|{\phi ,\psi }\rangle }$. The two-particle probability amplitudes are represented by

7 $$\begin{aligned} {\langle {\varphi ,\zeta |\phi ,\psi }\rangle }:= {\langle {\varphi |\phi }\rangle }{\langle {\zeta |\psi }\rangle } + \eta {\langle {\varphi |\psi }\rangle }{\langle {\zeta |\phi }\rangle }, \end{aligned}$$

where $\varphi ,\zeta$ are one-particle states of another global two-particle state vector and $\eta = 1$ for bosons and $\eta =-1$ for fermions. The right-hand side of Eq. (7) is symmetric if the one-particle state position is swapped with another, i.e., ${|{\phi ,\psi }\rangle }=\eta {|{\psi ,\phi }\rangle }$. From Eq. (7), the probability of finding two particles in the same state ${|{\varphi }\rangle }$ is ${\langle {\varphi ,\varphi |\phi ,\psi }\rangle } = (1+\eta ) {\langle {\varphi |\phi }\rangle }{\langle {\varphi |\psi }\rangle }$ which is zero for fermions due to Pauli exclusion principle^43^ and maximum for bosons. As Eq. (7) follows symmetry and linearity properties, the symmetric inner product of states with spaces of different dimensionality is defined as

8 $$\begin{aligned} {\langle {\psi _{k}}|}\cdot {|{\varphi _{1},\varphi _{2}}\rangle } \equiv {\langle {\psi _{k} \mid \varphi _{1},\varphi _{2}}\rangle } = {\langle {\psi _{k}|\varphi _{1}}\rangle }{|{\varphi _{2}}\rangle } + \eta {\langle {\psi _{k}|\varphi _{2}}\rangle }{|{\varphi _{1}}\rangle }, \end{aligned}$$

where ${|{{\tilde{\Phi }}}\rangle }={|{\varphi _{1},\varphi _{2}}\rangle }$ is the un-normalized state of two indistinguishable particles and ${|{\psi _{k}}\rangle }$ is a single-particle state. Equation (8) can be interpreted as a projective measurement where the two-particle un-normalized state ${|{{\tilde{\Phi }}}\rangle }$ is projected into a single particle state ${|{\psi _{k}}\rangle }$. Thus, the resulting normalized pure-state of a single particle after the projective measurement can be written as

9 $$\begin{aligned} {|{\phi _{k}}\rangle }=\frac{{\langle {\psi _{k}|\Phi }\rangle }}{\sqrt{{\langle {\Pi ^{(1)}_{k}}\rangle }}_{\Phi }}, \end{aligned}$$

where ${|{\Phi }\rangle }:=\frac{1}{\sqrt{{\mathbb {N}}}}{|{{\tilde{\Phi }}}\rangle }$ with ${\mathbb {N}}=1+\eta \mid {\langle {\varphi _{1}|\varphi _{2}}\rangle } \mid ^2$ and $\Pi ^{(1)}_{k}={|{\psi _{k}}\rangle } {\langle {\psi _{k}}|}$ is the one-particle projection operator. The one-particle identity operator can be defined as ${\mathbb {I}}^{(1)}:=\sum _{k}\Pi ^{(1)}_{k}$. So, using the linearity property of projection operators, one can write similar to Eq. (8):

10 $$\begin{aligned} {|{\psi _{k}}\rangle } {\langle {\psi _{k}}|} \cdot {|{\varphi _{1},\varphi _{2}}\rangle } = {\langle {\psi _{k}|\varphi _{1}}\rangle }{|{\psi _{k},\varphi _{2}}\rangle } + \eta {\langle {\psi _{k}|\varphi _{2}}\rangle }{|{\varphi _{1},\psi _{k}}\rangle }. \end{aligned}$$

Note that

11 $$\begin{aligned} {\mathbb {I}}^{(1)} {|{\Phi }\rangle } = 2 {|{\Phi }\rangle }, \end{aligned}$$

where the probability of resulting the state ${|{\psi _{k}}\rangle }$ is $p_{k}={\langle {\Pi ^{(1)}_{k}}\rangle }_{\Phi }/2$. The partial trace in this method can be written as

12 $$\begin{aligned}  \rho ^{(1)}&= \frac{1}{2} \text {Tr}^{(1)} {|{\Phi }\rangle } {\langle {\Phi }|} = \frac{1}{2} \sum _{k} {\langle {\psi _{k}|\Phi }\rangle } {\langle {\Phi |\psi _{k}}\rangle } = \sum _{k} p_{k} {|{\phi _{k}}\rangle } {\langle {\phi _{k}}|}, \end{aligned}$$

where the factor 1/2 comes from Eq. (11).

Another useful concept is that of *localized partial trace*^31^, which means that local measurements are being performed on a region of space *M* where the particle has a non-zero probability of being found. So, performing the localized partial trace on a region *M*, we get

13 $$\begin{aligned} \rho ^{(1)}_{M}=\frac{1}{{\mathbb {N}}_{M}}\text {Tr}^{(1)}_{M} {|{\Phi }\rangle } {\langle {\Phi }|}, \end{aligned}$$

where ${\mathbb {N}}_{M}$ is a normalization constant such that $\text {Tr}^{(1)}\rho ^{(1)}_{M}=1$. The entanglement entropy can be calculated as

14 $$\begin{aligned} E_{M}({|{\Phi }\rangle }):= S(\rho ^{(1)}_{M}) = -\sum _{i} \lambda _{i}\text {ln}\lambda _{i}, \end{aligned}$$

where $S(\rho )=-\text {Tr}(\rho \text {ln} \rho )$ is the von Neumann entropy and $\lambda _{i}$ are the eigenvalues of $\rho ^{(1)}_{M}$. We will call the state an entangled state if we get a non-zero value of Eq. (14).

### DoF trace-out for indistinguishable particles

In Ref.^37,65^, the authors have presented the DoF trace-out rule for two indistinguishable particles, each having two DoFs. Here, we generalize the DoF trace-out rule for two indistinguishable particles each having *n* DoFs from the general density matrix defined in Eq. (4) by substituting $p=2$. Suppose we want to trace-out the *j*-th DoF of location $s^x \in {\mathbb {S}}^{P}$. Then the reduced density matrix is calculated as

15 $$\begin{aligned}  \rho _{s^{x}_{{\bar{j}}}} \equiv&\text {Tr}_{s^{x}_{j}} \left( \rho ^{(2,n)} \right) \equiv \sum _{m_{j} \in {\mathbb {D}}_{j}} {\langle { s^x m_j \mid \rho ^{(2,n)} \mid s^x m_j }\rangle }\\ {}&:= \sum _{m_{j}} \bigg \lbrace \sum _{\begin{array}{c} \alpha ^1, \alpha ^2, a^{1}_{j}, a^{1}_{{\bar{j}}}, a^{2}_{1}, a^{2}_{2}, \ldots , a^{2}_{n} \\ \beta ^1, \beta ^2, b^{1}_{j}, b^{1}_{{\bar{j}}}, b^{2}_{1}, b^{2}_{2}, \ldots , b^{2}_{n} \end{array} } \kappa _p {\langle {s^x m_j \mid \alpha ^1 a^{1}_j}\rangle } {\langle {\beta ^1 b^{1}_{j} \mid s^x m_j}\rangle } {|{\alpha ^1 a^{1}_{{\bar{j}}}, \alpha ^2 a^{2}_{1} a^{2}_{2} \ldots a^{2}_{n} }\rangle }{\langle {\beta ^1 b^{1}_{{\bar{j}}}, \beta ^2 b^{2}_{1} b^{2}_{2} \ldots b^{2}_{n}}|} \\&\quad + \eta \sum _{\begin{array}{c} \alpha ^1, \alpha ^2, a^{1}_{1}, a^{1}_{2}, \ldots , b^{1}_{n} a^{2}_{j}, a^{2}_{{\bar{j}}}, \\ \beta ^1, \beta ^2, b^{1}_{j}, b^{1}_{{\bar{j}}}, b^{2}_{1}, b^{2}_{2} \end{array}, \ldots , b^{2}_{n} } {\langle {s^x m_j \mid \alpha ^2 a^{2}_j }\rangle } {\langle {\beta ^1 b^{1}_j \mid s^x m_j}\rangle } {|{\alpha ^1 a^{1}_{1} a^{1}_{2} \ldots a^{1}_{n} \alpha ^2 a^{2}_{{\bar{j}}}}\rangle } {\langle {\beta ^1 b^{1}_{{\bar{j}}}, \beta ^2 b^{2}_{1} b^{2}_{2} \ldots b^{2}_{n}}|} \\&\quad + \eta \sum _{\begin{array}{c} \alpha ^1, \alpha ^2, a^{1}_{j}, a^{1}_{{\bar{j}}}, a^{2}_{1}, a^{2}_{2}, \ldots , a^{2}_{n} \\ \beta ^1, \beta ^2, b^{1}_{1}, b^{1}_{2}, \ldots , b^{1}_{n} b^{2}_{j}, b^{2}_{{\bar{j}}} \end{array} } {\langle {s^x m_j \mid \alpha ^1 a^{1}_j}\rangle } {\langle {\beta ^2 b^{2}_j \mid s^x m_j}\rangle } {|{\alpha ^1 a^{1}_{{\bar{j}}}, \alpha ^2 a^{2}_{1} a^{2}_{2} \ldots a^{2}_{n}}\rangle }{\langle {\beta ^1 b^{1}_{1} b^{1}_{2} \ldots b^{1}_{n}, \beta ^2 b^{2}_{{\bar{j}}}}|} \\&\quad + \sum _{\begin{array}{c} \alpha ^1, \alpha ^2, a^{1}_{1}, a^{1}_{2}, \ldots , a^{1}_{n}, a^{2}_{j}, a^{2}_{{\bar{j}}}, \\ \beta ^1, \beta ^2, b^{1}_{1}, b^{1}_{2}, \ldots , b^{1}_{n} b^{2}_{j}, b^{2}_{{\bar{j}}} \end{array} } {\langle {s^x m_j \mid \alpha ^2 a^{2}_j }\rangle } {\langle {\beta ^2 b^{2}_j \mid s^x m_j}\rangle } {|{\alpha ^1 a^{1}_{1} a^{1}_{2} \ldots a^{1}_{n}, \alpha ^2 a^{2}_{{\bar{j}}}}\rangle } {\langle {\beta ^1 b^{1}_{1} b^{1}_{2} \ldots b^{1}_{n} \beta ^2 b^{2}_{{\bar{j}}}}|} \bigg \rbrace , \end{aligned}$$

where

$$\begin{aligned}  \kappa _p&=\kappa ^{\alpha ^1 \alpha ^2, \beta ^1, \beta ^2}_{ a^{1}_{j}, a^{1}_{{\bar{j}}}, a^{2}_{1}, a^{2}_{2}, \ldots , a^{2}_{n},b^{1}_{j}, b^{1}_{{\bar{j}}}, b^{2}_{1}, b^{2}_{2}, \ldots , b^{2}_{n}}, \hspace{1cm} \kappa _q= \kappa ^{\alpha ^1 \alpha ^2, \beta ^1, \beta ^2}_{a^{1}_{1}, a^{1}_{2}, \ldots , b^{1}_{n} a^{2}_{j}, a^{2}_{{\bar{j}}}, b^{1}_{j}, b^{1}_{{\bar{j}}}, b^{2}_{1}, b^{2}_{2}, \ldots , b^{2}_{n}},\\ \kappa _r&= \kappa ^{\alpha ^1 \alpha ^2, \beta ^1, \beta ^2}_{a^{1}_{j}, a^{1}_{{\bar{j}}}, a^{2}_{1}, a^{2}_{2}, \ldots , a^{2}_{n}, b^{1}_{1}, b^{1}_{2}, \ldots , b^{1}_{n}, b^{2}_{j}, b^{2}_{{\bar{j}}}}, \hspace{1cm} \kappa _s= \kappa ^{\alpha ^1 \alpha ^2, \beta ^1, \beta ^2}_{a^{1}_{1}, a^{1}_{2}, \ldots , a^{1}_{n}, a^{2}_{j}, a^{2}_{{\bar{j}}}, b^{1}_{1}, b^{1}_{2}, \ldots , b^{1}_{n}, b^{2}_{j}, b^{2}_{{\bar{j}}}}.\\ \end{aligned}$$

If we substitute $n=2$ in Eq. (15), then it reduces to the trace-out rule of^37^.

### How to calculate the concurrence between any two DoFs of two indistinguishable particles?

The calculation of the concurrence between any two DoFs from two spatial regions involves the following steps.

#### Step 1: applying the projector

In the general state given in Eq. (4), it is possible that each localized region has more than one particle. To calculate the concurrence, we have to ensure that each of the localized regions $s^1, s^2, \ldots , s^p$ has only one particle. For that, we have to apply a projector as follows.

Projecting $\rho ^{(p, n)}$ onto the operational subspace spanned by the basis

16 $$\begin{aligned} &{\mathscr {B}}^{s^1 s^2 \ldots s^p}\\ {}&= \lbrace {|{s^1 D^{1}_{1_{1}} \ldots D^{1}_{n_{1}}, s^2 D^{2}_{1_{1}} \ldots D^{2}_{n_{1}}, \ldots s^p D^{p}_{1_{1}} \ldots D^{p}_{n_{1}}}\rangle }, \\ {}&{|{s^1 D^{1}_{1_{2}} \ldots D^{1}_{n_{1}}, s^2 D^{2}_{1_{1}} \ldots D^{2}_{n_{1}}, \ldots s^p D^{p}_{1_{1}} \ldots D^{p}_{n_{1}}}\rangle }, \\&\vdots \\&{|{s^1 D^{1}_{1_{k_{1}}} \ldots D^{1}_{n_{k_{n}}}, s^2 D^{2}_{1_{k_{1}}}, \ldots s^p D^{p}_{1_{k_{1}}} D^{p}_{2_{k_{2}}} \ldots D^{p}_{n_{k_{n}}}}\rangle }\rbrace , \end{aligned}$$

by the projector

17 $$\begin{aligned} {\mathscr {P}}_{s^1 s^2 \ldots s^p}=\sum _{x^{i}_{j} \in {\mathbb {D}}_{j}, i \in {\mathbb {N}}_p, j \in {\mathbb {N}}_{n}} {|{s^{1} x^{1}_{1}x^{1}_{2} \ldots x^{1}_{n}, s^{2} x^{2}_{1}x^{2}_{2} \ldots x^{2}_{n}, \ldots , s^{p} x^{p}_{1}x^{p}_{2} \ldots x^{p}_{n} }\rangle } {\langle {s^{1} x^{1}_{1}x^{1}_{2} \ldots x^{1}_{n}, s^{2} x^{2}_{1}x^{2}_{2} \ldots x^{2}_{n}, \ldots , s^{p} x^{p}_{1}x^{p}_{2} \ldots x^{p}_{n} }|}, \end{aligned}$$

results in

18 $$\begin{aligned} \rho ^{(p, n)}_{s^1 s^2 \ldots s^p}=\dfrac{{\mathscr {P}}_{s^1 s^2 \ldots s^p} \rho ^{(p,n)} {\mathscr {P}}_{s^1 s^2 \ldots s^p}}{\text {Tr} \left( {\mathscr {P}}_{s^1 s^2 \ldots s^p} \rho ^{(p,n)} \right) }. \end{aligned}$$

It’s important to note that one might initially assume that applying a projection would limit the occupancy of the relevant modes to exactly one, seemingly defeating the purpose of using indistinguishable particles, as it would allow for proper labeling of particles by spatial modes. However, this assumption is not accurate. The projection operation encompasses all possible scenarios where each localized region contains one particle, as illustrated in Eq. (17). Consequently, even after the projection operation, it remains impossible to uniquely label the particles with the projected modes.

Therefore, it’s crucial to understand that the calculation of monogamy is not a mere consequence of the projection operation; rather, it serves the purpose of eliminating scenarios where no entanglement exists. The rationale behind employing the projection operation is to facilitate the calculation of entanglement between the number of localized regions, which corresponds to the number of particles involved. If any region contains more than one particle, it would lead to situations where other regions have no particles. Thus, calculating entanglement while including these regions would lack meaningful interpretation.

In summary, projections are employed for the sake of computational simplicity, and entanglement does not arise as a byproduct of this operation. It can be verified that, even without the projection operation, the entanglement calculation would yield the same results.

#### Step 2: tracing out non-contributing localized regions

To calculate the concurrence between two spatial regions, we have to trace out other $(p-2)$ regions using the method described in Eq. (15). The trace out rule for tracing out say $s^{h} \in {\mathbb {S}}^p$ region can be described as

19 $$\begin{aligned} &\rho ^{(p-1,n)}_{\left( {\mathbb {S}}^p - \lbrace s^{h} \rbrace \right) }=\text {Tr}_{s^{h}} \left( \rho ^{(p, n)} \right) =\sum _{m^{h}_{1},m^{h}_{2}, \ldots , m^{h}_{n}} {\langle {s^{h} m^{h}_{1} m^{h}_{2} \ldots m^{h}_{n} \mid \rho ^{(p, n)} \mid s^{h} m^{h}_{1} m^{h}_{2} \ldots m^{h}_{n} }\rangle }, \end{aligned}$$

where $m^{h}_{j}$ span ${\mathbb {D}}_{j}$ for $j \in {\mathbb {N}}_{n}$.

Thus if we trace out *k* number of particles from the localized regions $s^{h_{1}},s^{h_{1}}, \ldots , s^{h_{k}}$, then the reduced density matrix is represented as

20 $$\begin{aligned}  \rho ^{(p-h,n)}_{\left( {\mathbb {S}}^p - \lbrace s^{h_{1}},s^{h_{1}}, \ldots , s^{h_{k}}\rbrace \right) }&=\text {Tr}_{s^{h_{1}},s^{h_{2}}, \ldots , s^{h_{k}}} \left( \rho ^{(p, n)} \right) \\&= \sum _{ \begin{array}{c} s^{h_{i}} \in {\mathbb {S}}^p, m^{h_{i}}_{j} \in {\mathbb {D}}_{j} \end{array}} {\langle {s^{h_{1}} m^{h_{1}}_{1} m^{h_{1}}_{2} \ldots m^{h_{1}}_{n}, \ldots , s^{h_{k}} m^{h_{k}}_{1} m^{h_{k}}_{2} \ldots m^{h_{k}}_{n} \mid \rho ^{(p, n)} \mid s^{h_{1}} m^{h_{1}}_{1} m^{h_{1}}_{2} \ldots m^{h_{1}}_{n}, \ldots , s^{h_{k}} m^{h_{k}}_{1} m^{h_{k}}_{2} \ldots m^{h_{k}}_{n}}\rangle }. \end{aligned}$$

Suppose we want to calculate the concurrence between the particle in the location $s^r$ and the particle in the location $s^t$ where $s^r, s^t \in {\mathbb {S}}^p$, we apply the DoF trace-out rule as defined in Eq. (15). Thus the reduced density matrix is

21 $$\begin{aligned} \rho ^{\left( 2,n \right) }_{s^{r},s^{t}}=\text {Tr}_{ \left( {\mathbb {S}} -\lbrace s^{r}, s^{t}\rbrace \right) } \left( \rho ^{(p, n)} \right) . \end{aligned}$$

#### Step 3: tracing out non-contributing DoFs

To calculate the concurrence between the *v*-th DoF of the particle in the location $s^r$ and the *w*-th DoF of the particle in the location $s^t$ where $1\le v, w \le n$, we have to trace-out all the other non-contributing DoFs from these two locations using the DoF trace-out rule as defined in Eq. (15). So, the reduced density matrix of the *v*-th and the *w*-th DoF of the locations $s^r$ and $s^t$ respectively is given by

22 $$\begin{aligned}  \rho ^{\left( 2,1 \right) }_{s^{r}_{v},s^{t}_{w}}&= \text {Tr}_{\left( s^{r}_{{\bar{v}}},s^{t}_{{\bar{w}}}\right) } \left( \rho ^{\left( 2,n \right) }_{s^{r},s^{t}}\right) = \sum _{ m^{r}_{j}, m^{t}_{j} \in {\mathbb {D}}_{j}} {\langle { \psi ^{s^{r}}_{m_{{\bar{v}}}}, \psi ^{s^{t}}_{m_{{\bar{w}}}} \mid \rho ^{\left( 2,n \right) }_{s^{r},s^{t}} \mid \psi ^{s^{r}}_{m_{{\bar{v}}}}, \psi ^{s^{t}}_{m_{{\bar{w}}}} }\rangle }, \end{aligned}$$

where $${|{\psi ^{s^{r}}_{m_{{\bar{v}}}}}\rangle }={|{s^{r} m^{r}_{1}m^{r}_{2} \ldots m^{r}_{(v-1)} m^{r}_{(v+1)} \ldots m^{r}_{n}}\rangle }$$  and $${|{\psi ^{s^{t}}_{m_{{\bar{w}}}}}\rangle }={|{ s^{t} m^{t}_{1}m^{t}_{2} \ldots m^{t}_{(w-1)} m^{t}_{(w+1)} \ldots m^{t}_{n}}\rangle }$$.

#### Step 4: calculation of the eigenvalues

To calculate the concurrence of $\rho ^{\left( 2,1 \right) }_{s^{r}_{v}, s^{t}_{w}}$, i.e., ${\mathscr {C}}_{s^{r}_{v}\mid s^{t}_{w}}$, we have to calculate the following

23 $$\begin{aligned} {\widetilde{\rho }}_{s^{r}_{v},s^{t}_{w}}=\sigma ^{s^r}_{y} \otimes \sigma ^{s^t}_{y} \rho ^{*}_{s^{r}_{v},s^{t}_{w}} \sigma ^{s^r}_{y} \otimes \sigma ^{s^t}_{y}, \end{aligned}$$

where $\sigma ^{s^r}_{y} = {|{s^r}\rangle }{\langle {s^r}|} \otimes \sigma _{y}$, and similarly $\sigma ^{s^t}_{y} = {|{s^t}\rangle }{\langle {s^t}|} \otimes \sigma _{y}$, and $\sigma _{y}$ is Pauli matrix and the asterisk denotes complex conjugation.

Now we have to calculate the eigenvalues of the non-hermitian matrix

24 $$\begin{aligned} {\mathscr {R}}_{s^{r}_{v},s^{t}_{w}}=\rho _{s^{r}_{v},s^{t}_{w}} {\widetilde{\rho }}_{s^{r}_{v},s^{t}_{w}}. \end{aligned}$$

Finally, the concurrence is calculated as the

25 $$\begin{aligned} {\mathscr {C}}_{s^{r}_{v}\mid s^{t}_{w}}=\text {max} \left\{ 0, \sqrt{\lambda _{4}}-\sqrt{\lambda _{3}}-\sqrt{\lambda _{2}}-\sqrt{\lambda _{1}} \right\} , \end{aligned}$$

where $\lambda _{i}$’s are the eigenvalues of ${\mathscr {R}}_{s^{r}_{v}, s^{t}_{w}}$ in decreasing order.

### Proof of MoE for three indistinguishable particles each having a single DoF

Here, we calculate monogamy for three particles each having a single DoF, for example, spin DoF having eigenstates $\lbrace {|{\uparrow }\rangle }, {|{\downarrow }\rangle } \rbrace$ in three localized regions ${\mathbb {S}}^3$. Trivially, we can show that if each particle is in the same eigenstate of the same DoF, for example, ${|{\uparrow }\rangle }$ eigenstate in spin DoF, then the concurrence between any two particles between any two locations is zero.

Let us assume another situation where two particles are in the same eigenstate and the other particle is in the orthogonal eigenstate of the same DoF. Without loss of generality, consider two particles are in ${|{\uparrow }\rangle }$ eigenstate and the other is in ${|{\downarrow }\rangle }$ eigenstate in spin DoF. Thus the general state can be written as

26 $$\begin{aligned}  {|{\Psi ^{\left( 3,1 \right) }}\rangle }&=\sum _{\alpha ^{i} \in {\mathbb {S}}^3, i \in {\mathbb {N}}_3} \eta ^u \kappa ^{\alpha ^{1}, \alpha ^{2}, \alpha ^{3}}_{a^{1}, a^{2}, a^{3}} {|{\alpha ^{1} a^{1}, \alpha ^{2} a^{2}, \alpha ^{3} a^{3} }\rangle } \\&= \kappa ^{\alpha ^{1}, \alpha ^{2}, \alpha ^{3}}_{\uparrow , \uparrow ,\downarrow } {|{\alpha ^1 \uparrow , \alpha ^2 \uparrow , \alpha ^3 \downarrow }\rangle } + \eta \kappa ^{\alpha ^{1}, \alpha ^{2}, \alpha ^{3}}_{ \uparrow , \downarrow , \uparrow } {|{\alpha ^1 \uparrow , \alpha ^2 \downarrow , \alpha ^3 \uparrow }\rangle } + \kappa ^{\alpha ^{1}, \alpha ^{2}, \alpha ^{3}}_{\downarrow , \uparrow ,\uparrow } {|{\alpha ^1 \downarrow a^{1}_{2}, \alpha ^2 \uparrow a^{2}_{2}, \alpha ^3 \uparrow a^{3}_{2}}\rangle }.\\ \end{aligned}$$

Here $a^{i} \in \lbrace {|{\uparrow }\rangle }, {|{\downarrow }\rangle } \rbrace$, for $i \in \lbrace 1, 2, 3 \rbrace$ such that $a^{i} \ne a^{i^{\prime }}$ for all $i \ne i^{\prime }$ and if ${|{\uparrow }\rangle }=-\frac{1}{2}, {|{\downarrow }\rangle }=+\frac{1}{2}$ then $\sum a^{i} = -\frac{1}{2}$. The value of $\eta =0$ if $\left( \alpha ^{i} = \alpha ^{\prime }\right) \wedge \left( a^{i}=a^{i^{\prime }} \right)$ for all $i \ne i^{\prime }$.

The density matrix of Eq. (26) can be written as

27 $$ \begin{aligned}  \rho ^{\left( 3, 1\right) } = \sum _{\begin{array}{c} \alpha ^{i}, \beta ^{i} \in {\mathbb {S}}^3  \&  i \in {\mathbb {N}}_3 \end{array}} \eta ^{\left( u + {\bar{u}} \right) } \kappa ^{\alpha ^{1},\alpha ^{2},\alpha ^{3}}_{a^{1}, a^{2},a^{3}} \kappa ^{\beta ^{1}, \beta ^{2}, \beta ^{3}*}_{b^{1}, b^{2}, b^{3}} {|{\alpha ^{1} a^{1}, \alpha ^{2} a^{2}, \alpha ^{3} a^{3} }\rangle } {\langle {\beta ^{1} b^{1}, \beta ^{2} b^{2}, \beta ^{3} b^{3} }|}. \end{aligned}$$

Here $a^{i}, b^{i} \in \lbrace {|{\uparrow }\rangle }, {|{\downarrow }\rangle } \rbrace$, for $i \in \lbrace 1, 2, 3\rbrace$ such that $a^{i} \ne a^{i^{\prime }}$ and $b^{i} \ne b^{i^{\prime }}$ for all $i \ne i^{\prime }$. Also if we take ${|{\uparrow }\rangle }=-\frac{1}{2}, {|{\downarrow }\rangle }=+\frac{1}{2}$ then $\sum a^{i} =\sum b^{i}= -\frac{1}{2}$. The value of $\eta =0$ if $\left\{ \left( \alpha ^{i} = \alpha ^{\prime }\right) \vee \left( \beta ^{i} = \beta ^{\prime }\right) \right\} \rbrace \wedge \left\{ \left( a^{i} =a^{i^{\prime }} \right) \vee \left( b^{i} =b^{i^{\prime }} \right) \right\}$ for all $i \ne i^{\prime }$. The normalization condition in this case is

28 $$\begin{aligned} \sum _{\alpha ^{i}, \beta ^{i} \in {\mathbb {S}}^3, a^{i}, b^{i} \in \lbrace \uparrow , \downarrow \rbrace } \kappa ^{\alpha ^{1},\alpha ^{2},\alpha ^{3}}_{a^{1}, a^{2}, a^{3}} \kappa ^{\beta ^{1}, \beta ^{2}, \beta ^{3}*}_{b^{1}, b^{2}, b^{3}} =1, \end{aligned}$$

where $\alpha ^{i}=\beta ^{i}$, $a^{i}=b^{i}$ for all $i \in \lbrace 1, 2, 3 \rbrace$.

Now we calculate the concurrence by the steps described in “How to calculate the concurrence between any two DoFs of two indistinguishable particles?” section.

#### Step 1: applying the projector

Here, we have to apply the projector ${\mathscr {P}}^{\left( 3, 1\right) }_{s^1s^2s^3}$ in $\rho ^{\left( 3, 1\right) }$ so that in each of the location $s^{1}$, $s^{2}$, and $s^{3}$ have exactly one particle which is defined as

29 $$\begin{aligned}  {\mathscr {P}}^{\left( 3, 1\right) }_{s^1s^2s^3}&=\sum _{ \begin{array}{c} x^{i} \in \lbrace \uparrow , \downarrow \rbrace \end{array}} {|{s^{1} x^{1}, s^{2} x^{2}, s^{3} x^{3} }\rangle } {\langle {s^{1} x^{1}, s^{2} x^{2}, s^{3} x^{3} }|}. \end{aligned}$$

Thus after applying the projector, we get the density matrix as

30 $$\begin{aligned}  \rho ^{\left( 3,1 \right) }_{s^1s^2s^3}&= \frac{{\mathscr {P}}^{\left( 3,1 \right) }_{s^1s^2s^3} \rho ^{\left( 3, 1\right) } {\mathscr {P}}^{\left( 3,1 \right) }_{s^1s^2s^3}}{\text {Tr}\left( {\mathscr {P}}^{\left( 3,1 \right) }_{s^1s^2s^3} \rho ^{\left( 3, 1\right) } \right) } = \dfrac{ \sum _{h,k \in \lbrace 1,2,3\rbrace } \eta ^{\left( k-1 \right) } z_{h}z^{*}_{k}\rho ^{\left( 3,1\right) }_{hk} }{ \sum _{h \in \lbrace 1,2,3\rbrace } z_{h}z^{*}_{h} }, \end{aligned}$$

where the values of

31 $$\begin{aligned}  z_1&=\kappa ^{s^1,s^2,s^3}_{\uparrow ,\uparrow ,\downarrow }, \hspace{0.5cm} z_2=\kappa ^{s^1,s^2,s^3}_{\uparrow , \downarrow , \uparrow }, \hspace{0.5cm} z_3=\kappa ^{s^1,s^2,s^3}_{\downarrow , \uparrow ,\uparrow }, \end{aligned}$$

and the complex conjugates of $z_j$ for $j \in \lbrace 1, 2, 3\rbrace$ can be calculated accordingly. Also $\rho ^{\left( 3,1\right) }_{hk}={|{\psi }\rangle }^{(3,1)}_{h}{\langle {\psi }|}^{(3,1)}_{k}$ where

32 $$\begin{aligned}  {|{\psi }\rangle }^{(3,1)}_{1}&={|{s^{1} \uparrow , s^{2} \uparrow , s^{3} \downarrow }\rangle }, \hspace{0.2cm} {|{\psi }\rangle }^{(3,1)}_{2}={|{s^{1} \uparrow , s^{2} \downarrow , s^{3} \uparrow }\rangle }, \hspace{0.2cm} {|{\psi }\rangle }^{(3,1)}_{3}={|{s^{1} \downarrow , s^{2} \uparrow , s^{3} \uparrow }\rangle }, \end{aligned}$$

and the complex conjugates of ${|{\psi }\rangle }^{(3,1)}_{j}$ for $j \in \lbrace 1, 2, 3\rbrace$ can be calculated accordingly.

For the simplicity of the further calculations, we can expand Eq. (30) as

33 $$\begin{aligned}  \rho ^{\left( 3,1 \right) }_{s^1s^2s^3}&= z_1 z^{*}_1 \rho ^{\left( 3,1\right) }_{11} + \eta z_1 z^{*}_2 \rho ^{\left( 3,1\right) }_{12} + z_1 z^{*}_3 \rho ^{\left( 3,1\right) }_{13} + z_2 z^{*}_1 \rho ^{\left( 3,1\right) }_{21} + \eta z_2 z^{*}_2 \rho ^{\left( 3,1\right) }_{22} + z_2 z^{*}_3 \rho ^{\left( 3,1\right) }_{23} + z_3 z^{*}_1 \rho ^{\left( 3,1\right) }_{31} + \eta z_3 z^{*}_2 \rho ^{\left( 3,1\right) }_{32} + z_3 z^{*}_3 \rho ^{\left( 3,1\right) }_{33} \\&= z_1 z^{*}_1 {|{s^{1} \uparrow , s^{2} \uparrow , s^{3} \downarrow }\rangle } {\langle {s^{1} \uparrow , s^{2} \uparrow , s^{3} \downarrow }|} + \eta z_1 z^{*}_2 {|{s^{1} \uparrow , s^{2} \uparrow , s^{3} \downarrow }\rangle } {\langle {s^{1} \uparrow , s^{2} \downarrow , s^{3} \uparrow }|} + z_1 z^{*}_3 {|{s^{1} \uparrow , s^{2} \uparrow , s^{3} \downarrow }\rangle } {\langle {s^{1} \downarrow , s^{2} \uparrow , s^{3} \uparrow }|} \\&\quad + z_2 z^{*}_1 {|{s^{1} \uparrow , s^{2} \downarrow , s^{3} \uparrow }\rangle } {\langle {s^{1} \uparrow , s^{2} \uparrow , s^{3} \downarrow }|} + \eta z_2 z^{*}_2 {|{s^{1} \uparrow , s^{2} \downarrow , s^{3} \uparrow }\rangle } {\langle {s^{1} \uparrow , s^{2} \downarrow , s^{3} \uparrow }|} + z_2 z^{*}_3 {|{s^{1} \uparrow , s^{2} \downarrow , s^{3} \uparrow }\rangle } {\langle {s^{1} \downarrow , s^{2} \uparrow , s^{3} \uparrow }|} \\&\quad + z_3 z^{*}_1 {|{s^{1} \downarrow , s^{2} \uparrow , s^{3} \uparrow }\rangle } {\langle {s^{1} \uparrow , s^{2} \uparrow , s^{3} \downarrow }|} + \eta z_3 z^{*}_2 {|{s^{1} \downarrow , s^{2} \uparrow , s^{3} \uparrow }\rangle } {\langle {s^{1} \uparrow , s^{2} \downarrow , s^{3} \uparrow }|} + z_3 z^{*}_3 {|{s^{1} \downarrow , s^{2} \uparrow , s^{3} \uparrow }\rangle } {\langle {s^{1} \downarrow , s^{2} \uparrow , s^{3} \uparrow }|} \\&= \kappa ^{s^1,s^2,s^3}_{\uparrow ,\uparrow ,\downarrow }\kappa ^{s^1,s^2,s^3*}_{\uparrow ,\uparrow ,\downarrow } {|{s^{1} \uparrow , s^{2} \uparrow , s^{3} \downarrow }\rangle } {\langle {s^{1} \uparrow , s^{2} \uparrow , s^{3} \downarrow }|} + \eta \kappa ^{s^1,s^2,s^3}_{\uparrow ,\uparrow ,\downarrow }\kappa ^{s^1,s^2,s^3*}_{\uparrow , \downarrow , \uparrow } {|{s^{1} \uparrow , s^{2} \uparrow , s^{3} \downarrow }\rangle } {\langle {s^{1} \uparrow , s^{2} \downarrow , s^{3} \uparrow }|} \\&\quad + \kappa ^{s^1,s^2,s^3}_{\uparrow ,\uparrow ,\downarrow }\kappa ^{s^1,s^2,s^3*}_{\downarrow , \uparrow ,\uparrow } {|{s^{1} \uparrow , s^{2} \uparrow , s^{3} \downarrow }\rangle } {\langle {s^{1} \downarrow , s^{2} \uparrow , s^{3} \uparrow }|} \\&\quad + \kappa ^{s^1,s^2,s^3}_{\uparrow , \downarrow , \uparrow }\kappa ^{s^1,s^2,s^3*}_{\uparrow ,\uparrow ,\downarrow } {|{s^{1} \uparrow , s^{2} \downarrow , s^{3} \uparrow }\rangle } {\langle {s^{1} \uparrow , s^{2} \uparrow , s^{3} \downarrow }|} + \eta \kappa ^{s^1,s^2,s^3}_{\uparrow , \downarrow , \uparrow }\kappa ^{s^1,s^2,s^3*}_{\uparrow , \downarrow , \uparrow } {|{s^{1} \uparrow , s^{2} \downarrow , s^{3} \uparrow }\rangle } {\langle {s^{1} \uparrow , s^{2} \downarrow , s^{3} \uparrow }|} \\&\quad + \kappa ^{s^1,s^2,s^3}_{\uparrow , \downarrow , \uparrow }\kappa ^{s^1,s^2,s^3*}_{\downarrow , \uparrow ,\uparrow } {|{s^{1} \uparrow , s^{2} \downarrow , s^{3} \uparrow }\rangle } {\langle {s^{1} \downarrow , s^{2} \uparrow , s^{3} \uparrow }|} \\&\quad + \kappa ^{s^1,s^2,s^3}_{\downarrow , \uparrow ,\uparrow }\kappa ^{s^1,s^2,s^3*}_{\uparrow ,\uparrow ,\downarrow } {|{s^{1} \downarrow , s^{2} \uparrow , s^{3} \uparrow }\rangle } {\langle {s^{1} \uparrow , s^{2} \uparrow , s^{3} \downarrow }|} + \eta \kappa ^{s^1,s^2,s^3}_{\downarrow , \uparrow ,\uparrow } \kappa ^{s^1,s^2,s^3*}_{\uparrow , \downarrow , \uparrow } {|{s^{1} \downarrow , s^{2} \uparrow , s^{3} \uparrow }\rangle } {\langle {s^{1} \uparrow , s^{2} \downarrow , s^{3} \uparrow }|} \\&\quad + \kappa ^{s^1,s^2,s^3}_{\downarrow , \uparrow ,\uparrow } \kappa ^{s^1,s^2,s^3*}_{\downarrow , \uparrow ,\uparrow } {|{s^{1} \downarrow , s^{2} \uparrow , s^{3} \uparrow }\rangle } {\langle {s^{1} \downarrow , s^{2} \uparrow , s^{3} \uparrow }|}. \end{aligned}$$

It can be seen easily that the denominator of Eq. (30), i.e., $\sum _{h \in \lbrace 1,2,3\rbrace } z_{h}z^{*}_{h}=1$ according to Eq. (28).

#### Step 2: tracing out the region $s^3$

Now we have to trace out the particle at the region $s^3$. So, we get the reduced density matrix as

34 $$\begin{aligned}  \rho ^{\left( 2, 1\right) }_{s^1 s^2 }&=\text {Tr}_{s^3} \left( \rho ^{\left( 3, 1\right) }_{s^1 s^2 s^3 } \right) =\sum _{\begin{array}{c} m^{3} \in \lbrace \uparrow , \downarrow \rbrace \end{array}} {\langle {s^3 m^{3} \mid \rho ^{\left( 3, 1\right) }_{s^1 s^2 s^3 } \mid s^3 m^{3}}\rangle } = \dfrac{ \sum _{h,k \in \lbrace 1,2,3\rbrace } \eta ^{\left( k -1 \right) } z_{h}z^{*}_{k}\rho ^{\left( 2,1\right) }_{hk} }{ \sum _{h \in \lbrace 1,2,3\rbrace } z_{h}z^{*}_{h}}. \end{aligned}$$

The values of $\rho ^{\left( 2,1\right) }_{hk}={|{\psi }\rangle }^{(2,1)}_{h}{\langle {\psi }|}^{(2,1)}_{k}$ where

35 $$\begin{aligned}  {|{\psi }\rangle }^{(2,1)}_{1}&={|{s^{1} \uparrow , s^{2} \uparrow }\rangle }, \hspace{0.2cm} {|{\psi }\rangle }^{(2,1)}_{2}={|{s^{1} \uparrow , s^{2} \downarrow , }\rangle }, \hspace{0.2cm} {|{\psi }\rangle }^{(2,1)}_{3}={|{s^{1} \downarrow , s^{2} \uparrow }\rangle }, \hspace{0.2cm} {|{\psi }\rangle }^{(2,1)}_{4}={|{s^{1} \uparrow , s^{2} \uparrow }\rangle }, \\ \rho ^{\left( 2,1\right) }_{12}&=\rho ^{\left( 2,2\right) }_{13}=\rho ^{\left( 2,2\right) }_{21}=\rho ^{\left( 2,1\right) }_{31}=0, \end{aligned}$$

and the complex conjugates of ${|{\psi }\rangle }^{(2,1)}_{j}$ for $j \in \lbrace 1, 2, 3\rbrace$ can be calculated accordingly.

Now expanding Eq. (34), we get

36 $$\begin{aligned}  \rho ^{\left( 2,1 \right) }_{s^1s^2}&= z_1 z^{*}_1 \rho ^{\left( 2,1\right) }_{11} + \eta z_2 z^{*}_2 \rho ^{\left( 2,1\right) }_{22} + z_2 z^{*}_3 \rho ^{\left( 2,1\right) }_{23} + \eta z_3 z^{*}_2 \rho ^{\left( 2,1\right) }_{32} + z_3 z^{*}_3 \rho ^{\left( 2,1\right) }_{33}. \\ \end{aligned}$$

#### Step 3: calculation of the squared concurrence of $\rho ^{\left( 2, 1\right) }_{s^{1} s^{2}}$ denoted by ${\mathscr {C}}^{2}_{s^1 \mid s^2}$

To calculate concurrence for $\rho ^{\left( 2, 1\right) }_{s^{1} s^{2} }$, we have to calculate the following

37 $$\begin{aligned} {\widetilde{\rho }}^{\left( 2, 1\right) }_{s^{1} s^{2} }= \sigma ^{s^1}_{y} \otimes \sigma ^{s^2}_{y}\rho ^{\left( 2, 1\right) *}_{s^{1} s^{2} } \sigma ^{s^1}_{y} \otimes \sigma ^{s^2}_{y}, \end{aligned}$$

where $\sigma ^{s^1}_{y} = {|{s^1}\rangle }{\langle {s^1}|} \otimes \sigma _{y}$, $\sigma ^{s^2}_{y} = {|{s^2}\rangle }{\langle {s^2}|} \otimes \sigma _{y}$. Here $\sigma _{y}$ is the Pauli matrix and the asterisk denotes complex conjugation. The expression for $\sigma ^{s^1}_{y} \otimes \sigma ^{s^2}_{y}$ is

38 $$\begin{aligned}  \sigma ^{s^1}_{y} \otimes \sigma ^{s^2}_{y} = \rho ^{\left( 2,1\right) }_{23} + \rho ^{\left( 2,1\right) }_{32} - \rho ^{\left( 2,1\right) }_{41} - \rho ^{\left( 2,1\right) }_{14}. \end{aligned}$$

Thus the value of ${\widetilde{\rho }}^{\left( 2, 1\right) }_{s^{1} s^{2} }$ is

39 $$\begin{aligned}  {\widetilde{\rho }}^{\left( 2, 1\right) }_{s^{1} s^{2} } = |z_3|^2 \rho ^{\left( 2,1\right) }_{22} + \eta z_2 z^{*}_3\rho ^{\left( 2,1\right) }_{23} + z^{*}_2 z_3\rho ^{\left( 2,1\right) }_{32} + \eta |z_2|^2\rho ^{\left( 2,1\right) }_{33} + |z_1|^2\rho ^{\left( 2,1\right) }_{44} - \rho ^{\left( 2,1\right) }_{41} - \rho ^{\left( 2,1\right) }_{14}. \end{aligned}$$

Finally, we have to calculate the eigenvalues of

40 $$\begin{aligned}  {\mathscr {R}}=\rho ^{\left( 2, 1\right) }_{s^{1} s^{2} }{\widetilde{\rho }}^{\left( 2, 1\right) }_{s^{1} s^{2} }&= (1+\eta ) |z_2 z_3|^2 \rho ^{\left( 2,1\right) }_{22} + |z_3|^3 (z_2 + \eta z^{*}_2) \rho ^{\left( 2,1\right) }_{23} + (1+\eta ) |z_2|^3 z^{*}_3 \rho ^{\left( 2,1\right) }_{32} + (1+\eta )|z_2 z_3 |^2 \rho ^{\left( 2,1\right) }_{33}. \end{aligned}$$

So, the value of square of the concurrence ${\mathscr {C}}^{2}_{s^1 \mid s^2}$ can be calculated using Eq. (25) as

41 $$\begin{aligned}  {\mathscr {C}}^{2}_{s^1\mid s^2} = 2|z_2z_3|^2+z^{2}_2z^{*2}_3+z^{*2}_2 z^{2}_3 - 2|\eta z_2z^{*}_{3}z^{*}_2z_3-z^{2}_2z^{2}_3|^2. \end{aligned}$$

#### Step 4: calculation of the squared concurrence of $\rho ^{\left( 2, 1\right) }_{s^{1} s^{3} }$ denoted by ${\mathscr {C}}^{2}_{s^1 \mid s^3}$

Similarly, to calculate the squared concurrence ${\mathscr {C}}^{2}_{s^1 \mid s^3}$, the first step is to trace out the particle at the region $s^2$ from $\rho ^{\left( 3,1 \right) }_{s^1s^2s^3}$. So, we get the reduced density matrix as

42 $$\begin{aligned}  \rho ^{\left( 2, 1\right) }_{s^1 s^3 }&=\text {Tr}_{s^2} \left( \rho ^{\left( 3, 1\right) }_{s^1 s^2 s^3 } \right) =\sum _{\begin{array}{c} m^{2} \in \lbrace \uparrow , \downarrow \rbrace \end{array}} {\langle {s^2 m^{2} \mid \rho ^{\left( 3, 1\right) }_{s^1 s^2 s^3 } \mid s^2 m^{2}}\rangle } = \dfrac{ \sum _{h,k \in \lbrace 1,2,3\rbrace } \eta ^{\left( k-1 \right) } z_{h}z^{*}_{k}\rho ^{\left( 2,2\right) }_{hk} }{ \sum _{h \in \lbrace 1,2,3\rbrace } z_{h}z^{*}_{h}}. \end{aligned}$$

The values of $\rho ^{\left( 2,1\right) }_{hk}={|{\psi }\rangle }^{(2,1)}_{h}{\langle {\psi }|}^{(2,1)}_{k}$ where

43 $$\begin{aligned}  {|{\psi }\rangle }^{(2,1)}_{1}&={|{s^{1} \uparrow , s^{3} \uparrow }\rangle }, \hspace{0.2cm} {|{\psi }\rangle }^{(2,1)}_{2}={|{s^{1} \uparrow , s^{3} \downarrow , }\rangle }, \hspace{0.2cm} {|{\psi }\rangle }^{(2,1)}_{3}={|{s^{1} \downarrow , s^{3} \uparrow }\rangle }, \\ \rho ^{\left( 2,1\right) }_{12}&=\rho ^{\left( 2,2\right) }_{21}=\rho ^{\left( 2,2\right) }_{23}=\rho ^{\left( 2,1\right) }_{32}=0, \end{aligned}$$

and the complex conjugates of ${|{\psi }\rangle }^{(2,1)}_{j}$ for $j \in \lbrace 1, 2, 3\rbrace$ can be calculated accordingly.

Now following similar calculations as above we get square of the concurrence between $s^1$ and $s^3$ is

44 $$\begin{aligned}  {\mathscr {C}}^{2}_{s^1 \mid s^3} = 2|z_1z_3|^2+z^{2}_{1}z^{*2}_{3}+z^{*2}_{1}z^{2}_{3} - 2|\eta z_1z^{*}_{3}z^{*}_{1}z_3-z^{2}_{1}z^{2}_{3}|^2. \end{aligned}$$

#### Step 5: calculation of the monogamy relation

Thus the monogamy relation from Eqs. (41) and (44) can be written as

45 $$\begin{aligned} {\mathscr {C}}^{2}_{s^1\mid s^2}+{\mathscr {C}}^{2}_{s^1 \mid s^3} = 4(1-|z_3|^{2})|z_3|^{2} \le 1. \end{aligned}$$

If we further trace-out the particle at $s^2$ from Eq. (34), we get

46 $$\begin{aligned}  \rho ^{\left( 1, 1\right) }_{s^{1}}&= \sum _{ m^{2} \in {\mathbb {D}}_{2} } {\langle {s^2 m^{2} \mid \rho ^{\left( 2, 1\right) }_{s^{1} s^{2} } \mid s^2 m^{2} }\rangle } = \left( \mid z_1\mid ^2+|z_2|^2 \right) {|{s^1 \uparrow }\rangle }{\langle {s^1 \uparrow }|}+ |z_1|^3 {|{s^1 \downarrow }\rangle }{\langle {s^1 \downarrow }|}. \end{aligned}$$

Thus, as Eq. (26) is a pure state so, we have ${\mathscr {C}}^{2}_{s^1\mid s^2 s^3} = 4 \text {det}(\rho ^{\left( 1, 1\right) }_{s^{1}})= 4(1-|z_3|^{2})|z_3|^{2} \le 1$.

So, we get monogamy equality as

47 $$\begin{aligned} {\mathscr {C}}^{2}_{s^1 \mid s^2} + {\mathscr {C}}^{2}_{s^1 \mid s^3} = {\mathscr {C}}^{2}_{s^1 \mid s^2s^3}. \end{aligned}$$

### Proof of MoE for three indistinguishable particles each having two DoFs

Here, we calculate monogamy for three particles each having two DoFs, for example, spin and orbital angular momentum (OAM) DoFs having eigenstates $\lbrace {|{\uparrow }\rangle }, {|{\downarrow }\rangle } \rbrace$ and $\lbrace {|{+l}\rangle }, {|{-l}\rangle } \rbrace$ respectively in three localized regions ${\mathbb {S}}^3$. We describe the first five cases where one of the eigenstates of the DoFs contributes to entanglement, and the other non-contributing DoFs take arbitrary values. Then we consider the other cases where contributing DoFs for entanglement can be in an arbitrary superposition of their eigenstates.

*Case 1* Each particle is in the same eigenstate of the same DoF, for example, ${|{\uparrow }\rangle }$ eigenstate in spin DoF. Trivial calculations show that the concurrence between any two particles between any two locations is zero.

*Case 2* Two particles are in the same eigenstate and the other particle is in the orthogonal eigenstate of the same DoF. Without loss of generality, consider two particles are in ${|{\uparrow }\rangle }$ eigenstate and the other is in ${|{\downarrow }\rangle }$ eigenstate in spin DoF. We take two DoFs in this case as the calculations are same for three DoFs.

Here, we calculate the monogamy of entanglement using three indistinguishable particles each having two DoFs, which are localized in three spatial regions $s^1$, $s^2$, and $s^3$ which we denote as ${\mathbb {S}}^3$. We consider two particles with ${|{\uparrow }\rangle }$ eigenstate and one particle with ${|{\downarrow }\rangle }$ eigenstate in their spin DoF as we calculate entanglement with only spin DoF. The other DoF of each particle can take any arbitrary eigenvalues. Thus the general state can be written as

48 $$\begin{aligned}  {|{\Psi ^{\left( 3,2 \right) }}\rangle }&=\sum _{\alpha ^{i} \in {\mathbb {S}}^3, i \in {\mathbb {N}}_3} \eta ^u \kappa ^{\alpha ^{1}, \alpha ^{2}, \alpha ^{3}}_{a^{1}_{1} a^{1}_{2}, a^{2}_{1} a^{2}_{2}, a^{3}_{1} a^{3}_{2}} {|{\alpha ^{1} a^{1}_{1} a^{1}_{2}, \alpha ^{2} a^{2}_{1} a^{2}_{2}, \alpha ^{3} a^{3}_{1} a^{3}_{2} }\rangle } \\&= \sum _{a^{i}_{2} \in {\mathbb {D}}_{2}} \eta ^{u_{2}} \kappa ^{\alpha ^{1}, \alpha ^{2}, \alpha ^{3}}_{\uparrow a^{1}_{2}, \uparrow a^{2}_{2},\downarrow a^{3}_{2}} {|{\alpha ^1 \uparrow a^{1}_{2}, \alpha ^2 \uparrow a^{2}_{2}, \alpha ^3 \downarrow a^{3}_{2}}\rangle } + \sum _{a^{i}_{2} \in {\mathbb {D}}_{2}} \eta ^{(1+u_{2})} \kappa ^{\alpha ^{1}, \alpha ^{2}, \alpha ^{3}}_{ \uparrow a^{1}_{2}, \downarrow a^{2}_{2}, \uparrow a^{3}_{2}} {|{\alpha ^1 \uparrow a^{1}_{2}, \alpha ^2 \downarrow a^{2}_{2}, \alpha ^3 \uparrow a^{3}_{2}}\rangle }\\&\quad + \sum _{a^{i}_{2} \in {\mathbb {D}}_{2}} \eta ^{u_{2}} \kappa ^{\alpha ^{1}, \alpha ^{2}, \alpha ^{3}}_{\downarrow a^{1}_{2}, \uparrow a^{2}_{2},\uparrow a^{3}_{2}} {|{\alpha ^1 \downarrow a^{1}_{2}, \alpha ^2 \uparrow a^{2}_{2}, \alpha ^3 \uparrow a^{3}_{2}}\rangle }. \end{aligned}$$

Here $a^{i}_{1} \in \lbrace {|{\uparrow }\rangle }, {|{\downarrow }\rangle } \rbrace$, $a^{i}_{2} \in {\mathbb {D}}_{2}$ for $i \in \lbrace 1, 2, 3 \rbrace$ such that $a^{i}_{1} \ne a^{i^{\prime }}_{1}$ for all $i \ne i^{\prime }$ and if ${|{\uparrow }\rangle }=-\frac{1}{2}, {|{\downarrow }\rangle }=+\frac{1}{2}$ then $\sum a^{i}_{1} = -\frac{1}{2}$. The value of $\eta =0$ if $\left( \alpha ^{i} = \alpha ^{\prime }\right) \wedge \left( a^{i}_{j}=a^{i^{\prime }}_{j} \right)$ for all $i \ne i^{\prime }$ where $j \in {\mathbb {N}}_2$.

The density matrix of Eq. (48) can be written as

49 $$ \begin{aligned}  \rho ^{\left( 3, 2\right) }&=\sum _{\begin{array}{c} \alpha ^{i}, \beta ^{i} \in {\mathbb {S}}^3  \&  i \in {\mathbb {N}}_3 \end{array}} \eta ^{\left( u + {\bar{u}} \right) } \kappa ^{\alpha ^{1},\alpha ^{2},\alpha ^{3}}_{a^{1}_{1} a^{1}_{2}, a^{2}_{1} a^{2}_{2},a^{3}_{1} a^{3}_{2}} \kappa ^{\beta ^{1}, \beta ^{2}, \beta ^{3}*}_{b^{1}_{1} b^{1}_{2},b^{2}_{1} b^{2}_{2},b^{3}_{1} b^{3}_{2}} {|{\alpha ^{1} a^{1}_{1} a^{1}_{2}, \alpha ^{2} a^{2}_{1} a^{2}_{2}, \alpha ^{3} a^{3}_{1} a^{3}_{2} }\rangle } {\langle {\beta ^{1} b^{1}_{1} b^{1}_{2}, \beta ^{2} b^{2}_{1} b^{2}_{2}, \beta ^{3} b^{3}_{1} b^{3}_{2} }|}. \\ \end{aligned}$$

Here $a^{i}_{1}, b^{i}_{1} \in \lbrace {|{\uparrow }\rangle }, {|{\downarrow }\rangle } \rbrace$, $a^{i}_{2}, b^{i}_{2} \in {\mathbb {D}}_{2}$ for $i \in \lbrace 1, 2, 3\rbrace$ such that $a^{i}_{1} \ne a^{i^{\prime }}_{1}$ and $b^{i}_{1} \ne b^{i^{\prime }}_{1}$ for all $i \ne i^{\prime }$. Also if we take ${|{\uparrow }\rangle }=-\frac{1}{2}, {|{\downarrow }\rangle }=+\frac{1}{2}$ then $\sum a^{i}_{1} =\sum b^{i}_{1}= -\frac{1}{2}$. The value of $\eta =0$ if $\left\{ \left( \alpha ^{i} = \alpha ^{\prime }\right) \vee \left( \beta ^{i} = \beta ^{\prime }\right) \right\} \rbrace \wedge \left\{ \left( a^{i}_{j}=a^{i^{\prime }}_{j} \right) \vee \left( b^{i}_{j}=b^{i^{\prime }}_{j} \right) \right\}$ for all $i \ne i^{\prime }$ where $j \in {\mathbb {N}}_2$. Here the normalization condition is

50 $$\begin{aligned} \sum _{\alpha ^{i}, \beta ^{i} \in {\mathbb {S}}^3, a^{i}_{1}, b^{i}_{1} \in \lbrace \uparrow , \downarrow \rbrace , a^{i}_{2}, b^{i}_{2} \in {\mathbb {D}}_{2}} \kappa ^{\alpha ^{1},\alpha ^{2},\alpha ^{3}}_{a^{1}_{1} a^{1}_{2}, a^{2}_{1} a^{2}_{2},a^{3}_{1} a^{3}_{2}} \kappa ^{\beta ^{1}, \beta ^{2}, \beta ^{3}*}_{b^{1}_{1} b^{1}_{2},b^{2}_{1} b^{2}_{2},b^{3}_{1} b^{3}_{2}} =1, \end{aligned}$$

where $\alpha ^{i}=\beta ^{i}$, $a^{i}_{j}=b^{i}_{j}$ for all $i \in \lbrace 1, 2, 3 \rbrace$ and $j \in \lbrace 1, 2 \rbrace$.

Now we calculate the concurrence by the steps described in “How to calculate the concurrence between any two DoFs of two indistinguishable particles?” section.

#### Step 1: applying the projector

First, we have to apply the projector ${\mathscr {P}}_{s^1s^2s^3}$ so that in each of the location $s^{1}$, $s^{2}$, and $s^{3}$ have exactly one particle which is defined as

51 $$\begin{aligned}  {\mathscr {P}}_{s^1s^2s^3}&=\sum _{ \begin{array}{c} x^{i}_{1} \in \lbrace \uparrow , \downarrow \rbrace , x^{i}_{2} \in {\mathbb {D}}_{2} \end{array}} {|{s^{1} x^{1}_{1} x^{1}_{2}, s^{2} x^{2}_{1} x^{2}_{2}, s^{3} x^{3}_{1} x^{3}_{2} }\rangle } {\langle {s^{1} x^{1}_{1} x^{1}_{2}, s^{2} x^{2}_{1} x^{2}_{2}, s^{3} x^{3}_{1} x^{3}_{2} }|}. \end{aligned}$$

Thus after applying the projector, we get the density matrix as

52 $$\begin{aligned}  \rho ^{\left( 3,2 \right) }_{s^1s^2s^3}&= \frac{{\mathscr {P}}_{s^1s^2s^3} \rho ^{\left( 3, 2\right) } {\mathscr {P}}_{s^1s^2s^3}}{\text {Tr}\left( {\mathscr {P}}_{s^1s^2s^3} \rho ^{\left( 3, 2\right) } \right) } =\sum _{\begin{array}{c} a^{i}_{2}, b^{i}_{2},x^{i}_{2} \in {\mathbb {D}}_{2} \end{array} } \dfrac{ \sum _{h,k \in \lbrace 1,2,3\rbrace } \eta ^{\left( k+ u_2 + \bar{u_2}-1 \right) } z_{h}z^{*}_{k}\rho ^{\left( 3,2\right) }_{hk} }{ \sum _{h \in \lbrace 1,2,3\rbrace } z_{h}z^{*}_{h} }, \end{aligned}$$

where $a^{i}_{2}=b^{i}_{2}=x^{i}_{2}$. The values of

53 $$\begin{aligned}  z_1&=\kappa ^{s^1,s^2,s^3}_{\uparrow a^{1}_{2},\uparrow a^{2}_{2},\downarrow a^{3}_{2}}, \hspace{0.2cm} z_2&=\kappa ^{s^1,s^2,s^3}_{\uparrow a^{1}_{2}, \downarrow a^{2}_{2}, \uparrow a^{3}_{2}}, \hspace{0.2cm} z_3&=\kappa ^{s^1,s^2,s^3}_{\downarrow a^{1}_{2},\uparrow a^{2}_{2},\uparrow a^{3}_{2}}, \end{aligned}$$

and the complex conjugates of $z_j$ for $j \in \lbrace 1, 2, 3\rbrace$ can be calculated accordingly.

Also $$\rho ^{\left( 3,2\right) }_{hk}={|{\psi }\rangle }^{(3,2)}_{h}{\langle {\psi }|}^{(3,2)}_{k}$$ where

54 $$\begin{aligned}  {|{\psi }\rangle }^{(3,2)}_{1}&={|{s^{1} \uparrow x^{1}_{2}, s^{2} \uparrow x^{2}_{2}, s^{3} \downarrow x^{3}_{2} }\rangle }, \\ {|{\psi }\rangle }^{(3,2)}_{2}&={|{s^{1} \uparrow x^{1}_{2}, s^{2} \downarrow x^{2}_{2}, s^{3} \uparrow x^{3}_{2} }\rangle }, \\ {|{\psi }\rangle }^{(3,2)}_{3}&={|{s^{1} \downarrow x^{1}_{2}, s^{2} \uparrow x^{2}_{2}, s^{3} \uparrow x^{3}_{2} }\rangle }, \end{aligned}$$

and the complex conjugates of ${|{\psi }\rangle }^{(3,2)}_{j}$ for $j \in \lbrace 1, 2, 3\rbrace$ can be calculated accordingly.

#### Step 2: tracing out the region $s^3$

Now we have to trace out the particle at the region $s^3$. So, we get the reduced density matrix as

55 $$\begin{aligned}  \rho ^{\left( 2, 2\right) }_{s^1 s^2 }&=\text {Tr}_{s^3} \left( \rho ^{\left( 3, 2\right) }_{s^1 s^2 s^3 } \right) =\sum _{\begin{array}{c} m^{3}_{1}, m^{3}_{1} \in \lbrace \uparrow , \downarrow \rbrace , m^{3}_{2} \in {\mathbb {D}}_{2} \end{array}} {\langle {s^3 m^{3}_{1} m^{3}_{2} \mid \rho ^{\left( 3, 2\right) }_{s^1 s^2 s^3 } \mid s^3 m^{3}_{1} m^{3}_{2}}\rangle } \\&= \sum _{\begin{array}{c} a^{i}_{2}, b^{i}_{2},x^{i}_{2} \in {\mathbb {D}}_{2} \end{array}} \dfrac{ \sum _{h,k \in \lbrace 1,2,3\rbrace } \eta ^{\left( k+ u_2 + \bar{u_2}-1 \right) } z_{h}z^{*}_{k}\rho ^{\left( 2,2\right) }_{hk} }{ \sum _{h \in \lbrace 1,2,3\rbrace } z_{h}z^{*}_{h}}, \end{aligned}$$

where $$a^{i}_{2}=b^{i}_{2}=x^{i}_{2}$$, and $$m^{3}_{2}=x^{3}_{2} $$. The values of $$\rho ^{\left( 2,2\right) }_{hk}={|{\psi }\rangle }^{(2,2)}_{h}{\langle {\psi }|}^{(2,2)}_{k}$$ where

56 $$\begin{aligned}  {|{\psi }\rangle }^{(2,2)}_{1}&={|{s^{1} \uparrow x^{1}_{2}, s^{2} \uparrow x^{2}_{2} }\rangle }, \hspace{0.2cm} {|{\psi }\rangle }^{(2,2)}_{2}={|{s^{1} \uparrow x^{1}_{2}, s^{2} \downarrow x^{2}_{2}, }\rangle }, \hspace{0.2cm} {|{\psi }\rangle }^{(2,2)}_{3}={|{s^{1} \downarrow x^{1}_{2}, s^{2} \uparrow x^{2}_{2}}\rangle }, \\ \rho ^{\left( 2,2\right) }_{12}&=\rho ^{\left( 2,2\right) }_{13}=\rho ^{\left( 2,2\right) }_{21}=\rho ^{\left( 2,2\right) }_{31}=0, \end{aligned}$$

and the complex conjugates of ${|{\psi }\rangle }^{(2,2)}_{j}$ for $j \in \lbrace 1, 2, 3\rbrace$ can be calculated accordingly.

#### Step 3: tracing out the second DoF

Finally tracing out the second DoF of each particle we have

57 $$\begin{aligned}  \rho ^{\left( 2, 1\right) }_{s^{1}_1 s^{2}_{1} }&= \sum _{ m^{1}_{2}, m^{2}_{2} \in {\mathbb {D}}_{2} } {\langle {s^1 m^{1}_{2}, s^2 m^{2}_{2} \mid \rho ^{\left( 2, 2\right) }_{s^{1}s^{2}} \mid s^1 m^{1}_{2}, s^2 m^{2}_{2}}\rangle } = \sum _{\begin{array}{c} a^{i}_{2}, b^{i}_{2},x^{i}_{2} \in {\mathbb {D}}_{2} \end{array}} \dfrac{ \sum _{h,k \in \lbrace 1,2,3\rbrace } \eta ^{\left( k+ u_2 + \bar{u_2}-1 \right) } z_{h}z^{*}_{k}\rho ^{\left( 2,1\right) }_{hk} }{ \sum _{h \in \lbrace 1,2,3\rbrace } z_{h}z^{*}_{h}}, \end{aligned}$$

where $a^{i}_{2}=b^{i}_{2}=x^{i}_{2}= m^{i}_{2}$. The values of $\rho ^{\left( 2,1\right) }_{hk}={|{\psi }\rangle }^{(2,1)}_{h}{\langle {\psi }|}^{(2,1)}_{k}$ where

58 $$\begin{aligned}  {|{\psi }\rangle }^{(2,1)}_{1}&={|{s^{1} \uparrow , s^{2} \uparrow }\rangle }, \hspace{0.2cm} {|{\psi }\rangle }^{(2,1)}_{2}={|{s^{1} \uparrow , s^{2} \downarrow }\rangle }, \hspace{0.2cm} {|{\psi }\rangle }^{(2,1)}_{3}={|{s^{1} \downarrow , s^{2} \uparrow }\rangle }, \\ \rho ^{\left( 2,1\right) }_{12}&=\rho ^{\left( 2,1\right) }_{13}=\rho ^{\left( 2,1\right) }_{21}=\rho ^{\left( 2,1\right) }_{31}=0, \end{aligned}$$

and the complex conjugates of ${|{\psi }\rangle }^{(2,1)}_{j}$ for $j \in \lbrace 1, 2, 3\rbrace$ can be calculated accordingly.

#### Step 4: calculation of the squared concurrence of $\rho ^{\left( 2, 1\right) }_{s^{1}_1 s^{2}_{1} }$

To calculate concurrence for $\rho ^{\left( 2, 1\right) }_{s^{1}_1 s^{2}_{1} }$, we have to calculate the following

59 $$\begin{aligned} {\widetilde{\rho }}^{\left( 2, 1\right) }_{s^{1}_1 s^{2}_{1} }= \sigma ^{s^1}_{y} \otimes \sigma ^{s^2}_{y}\rho ^{\left( 2, 1\right) *}_{s^{1}_1 s^{2}_{1} } \sigma ^{s^1}_{y} \otimes \sigma ^{s^2}_{y}, \end{aligned}$$

where $\sigma ^{s^1}_{y} = {|{s^1}\rangle }{\langle {s^1}|} \otimes \sigma _{y}$, $\sigma ^{s^2}_{y} = {|{s^2}\rangle }{\langle {s^2}|} \otimes \sigma _{y}$. Here $\sigma _{y}$ is the Pauli matrix and the asterisk denotes complex conjugation. Finally, we have to calculate the eigenvalues of ${\mathscr {R}}=\rho ^{\left( 2, 1\right) }_{s^{1}_1 s^{2}_{1} }{\widetilde{\rho }}^{\left( 2, 1\right) }_{s^{1}_1 s^{2}_{1} }$.

So, the value of square of the concurrence ${\mathscr {C}}^{2}_{s^1 \mid s^2}$ is

60 $$\begin{aligned}  {\mathscr {C}}^{2}_{s^1\mid s^2} = 2|z_2z_3|^2+z^{2}_2z^{*2}_3+z^{*2}_2 z^{2}_3 - 2|z_2z^{*}_{3}z^{*}_2z_3-z^{2}_2z^{2}_3|^2. \end{aligned}$$

#### Step 5: calculation of the squared concurrence ${\mathscr {C}}^{2}_{s^1 \mid s^3}$

Similarly, to calculate the squared concurrence ${\mathscr {C}}^{2}_{s^1 \mid s^3}$, the first step is to trace out the particle at the region $s^2$ from $\rho ^{\left( 3,2 \right) }_{s^1s^2s^3}$ as shown in Eq. (52). So, we get the reduced density matrix as

61 $$\begin{aligned}  \rho ^{\left( 2, 2\right) }_{s^1 s^3 }&=\text {Tr}_{s^2} \left( \rho ^{\left( 3, 2\right) }_{s^1 s^2 s^3 } \right) =\sum _{\begin{array}{c} m^{2}_{1} \in \lbrace \uparrow , \downarrow \rbrace , m^{2}_{2} \in {\mathbb {D}}_{2} \end{array}} {\langle {s^2 m^{2}_{1} m^{2}_{2} \mid \rho ^{\left( 3, 2\right) }_{s^1 s^2 s^3 } \mid s^2 m^{2}_{1} m^{2}_{2}}\rangle } \\&= \sum _{\begin{array}{c} a^{i}_{2}, b^{i}_{2},x^{i}_{2} \in {\mathbb {D}}_{2} \end{array}} \dfrac{ \sum _{h,k \in \lbrace 1,2,3\rbrace } \eta ^{\left( k+ u_2 + \bar{u_2}-1 \right) } z_{h}z^{*}_{k}\rho ^{\left( 2,2\right) }_{hk} }{ \sum _{h \in \lbrace 1,2,3\rbrace } z_{h}z^{*}_{h}}, \end{aligned}$$

where $$a^{i}_{2}=b^{i}_{2}=x^{i}_{2}$$, and $$m^{2}_{2}=x^{2}_{2} $$. The values of $$\rho ^{\left( 2,2\right) }_{hk}={|{\psi }\rangle }^{(2,2)}_{h}{\langle {\psi }|}^{(2,2)}_{k}$$ where

62 $$\begin{aligned}  {|{\psi }\rangle }^{(2,2)}_{1}&={|{s^{1} \uparrow x^{1}_{2}, s^{3} \uparrow x^{3}_{2} }\rangle }, \hspace{0.2cm} {|{\psi }\rangle }^{(2,2)}_{2}={|{s^{1} \uparrow x^{1}_{2}, s^{3} \downarrow x^{3}_{2}, }\rangle }, \hspace{0.2cm} {|{\psi }\rangle }^{(2,2)}_{3}={|{s^{1} \downarrow x^{1}_{2}, s^{3} \uparrow x^{3}_{2}}\rangle }, \\ \rho ^{\left( 2,2\right) }_{12}&=\rho ^{\left( 2,2\right) }_{21}=\rho ^{\left( 2,2\right) }_{23}=\rho ^{\left( 2,2\right) }_{32}=0, \end{aligned}$$

and the complex conjugates of ${|{\psi }\rangle }^{(2,2)}_{j}$ for $j \in \lbrace 1, 2, 3\rbrace$ can be calculated accordingly.

Now following similar calculations as above we get the square of the concurrence between $s^1$ and $s^3$ is

63 $$\begin{aligned}  {\mathscr {C}}^{2}_{s^1 \mid s^3} = 2|z_1z_3|^2+z^{2}_{1}z^{*2}_{3}+z^{*2}_{1}z^{2}_{3} - 2|z_1z^{*}_{3}z^{*}_{1}z_3-z^{2}_{1}z^{2}_{3}|^2. \end{aligned}$$

#### Step 6: calculation of the monogamy relation

Thus the monogamy relation from Eqs. (60) and (63) can be written as

64 $$\begin{aligned} {\mathscr {C}}^{2}_{s^1\mid s^2}+{\mathscr {C}}^{2}_{s^1 \mid s^3} = 4(1-|z_3|^{2})|z_3|^{2} \le 1. \end{aligned}$$

If we further trace-out the particle at $s^2$ from Eq. (57), we get

65 $$\begin{aligned}  \rho ^{\left( 1, 1\right) }_{s^{1}_1}&= \sum _{ m^{2}_{1} \in {\mathbb {D}}_{2} } {\langle {s^2 m^{2}_{1} \mid \rho ^{\left( 2, 1\right) }_{s^{1}_1,s^{2}_{1} } \mid s^2 m^{2}_{1} }\rangle } = \left( \mid z_1\mid ^2+|z_2|^2 \right) {|{s^1 \uparrow }\rangle }{\langle {s^1 \uparrow }|}+ |z_1|^3 {|{s^1 \downarrow }\rangle }{\langle {s^1 \downarrow }|}. \end{aligned}$$

Thus, as Eq. (48) is a pure state so, we have ${\mathscr {C}}^{2}_{s^1\mid s^2 s^3} = 4 \text {det}(\rho ^{\left( 1, 1\right) }_{s^{1}_1})= 4(1-|z_3|^{2})|z_3|^{2} \le 1$.

So, we get the monogamy equality as

66 $$\begin{aligned} {\mathscr {C}}^{2}_{s^1 \mid s^2} + {\mathscr {C}}^{2}_{s^1 \mid s^3} = {\mathscr {C}}^{2}_{s^1 \mid s^2s^3}. \end{aligned}$$

*Case 3* Two particles are in the same eigenstate in the same DoF (say ${|{\uparrow }\rangle }$ in spin) and the other particle is in a different eigenstate of another DoF (say ${|{+l}\rangle }$ in OAM). If the particles in the three regions ${\mathbb {S}}^3$ are measured in spin, spin, and OAM DoFs, then calculations reveal that

67 $$\begin{aligned} {\mathscr {C}}^{2}_{s^1|s^2} = {\mathscr {C}}^{2}_{s^1|s^3} = {\mathscr {C}}^{2}_{s^1|s^2s^3}=0. \end{aligned}$$

*Case 4* Two particles are in orthogonal eigenstate in the same DoF (say ${|{\uparrow }\rangle }$ and ${|{\downarrow }\rangle }$ in spin) and other particles is in different eigenstate of another DoF (say ${|{+l}\rangle }$ in OAM). By similar calculations as case 3, we get ${\mathscr {C}}^{2}_{s^1|s^2} \ne 0$, ${\mathscr {C}}^{2}_{s^1|s^3}=0$, and ${\mathscr {C}}^{2}_{s^1|s^2s^3}={\mathscr {C}}^{2}_{s^1|s^2}$ as follows.

Consider two particles with spin DoF having ${|{\uparrow }\rangle }$ and ${|{\downarrow }\rangle }$ eigenstates respectively and one particle with orbital angular momentum DoF with ${|{+l}\rangle }$ eigenstate. The eigenvalues of spin DoF and OAM DoF are represented by $a^{i}_{1} \in {\mathbb {D}}_{1}= \lbrace {|{\uparrow }\rangle }, {|{\downarrow }\rangle } \rbrace$ and $a^{i}_{2} \in {\mathbb {D}}_{2} =\lbrace {|{+l}\rangle }, {|{-l}\rangle }\rbrace$ respectively where $i \in \lbrace 1, 2, 3 \rbrace$. The other non-contributing DoFs in the entanglement of each particle can take any arbitrary eigenvalues. Thus the general state can be written as

68 $$\begin{aligned}  {|{\Psi ^{\left( 3,2 \right) }}\rangle }&= \sum _{a^{3}_{1} \in {\mathbb {D}}_{1},a^{1}_{2}, a^{2}_{2} \in {\mathbb {D}}_{2}} \eta ^{0} \kappa ^{\alpha ^{1}, \alpha ^{2}, \alpha ^{3}}_{\uparrow a^{1}_{2}, \downarrow a^{2}_{2}, a^{3}_{1} +l} {|{\alpha ^1 \uparrow a^{1}_{2}, \alpha ^2 \downarrow a^{2}_{2}, \alpha ^3 a^{3}_{1} +l}\rangle } \\ {}&\quad + \sum _{a^{2}_{1} \in {\mathbb {D}}_{1},a^{1}_{2}, a^{3}_{2} \in {\mathbb {D}}_{2}} \eta ^{1} \kappa ^{\alpha ^{1}, \alpha ^{2}, \alpha ^{3}}_{ \uparrow a^{1}_{2}, a^{2}_{1} +l, \downarrow a^{3}_{2}} {|{\alpha ^1 \uparrow a^{1}_{2}, \alpha ^2 a^{2}_{1} +l, \alpha ^3 \downarrow a^{3}_{2}}\rangle } \\&\quad + \sum _{a^{3}_{1} \in {\mathbb {D}}_{1},a^{1}_{2}, a^{2}_{2} \in {\mathbb {D}}_{2}} \eta ^{2} \kappa ^{\alpha ^{1}, \alpha ^{2}, \alpha ^{3}}_{\downarrow a^{1}_{2}, \uparrow a^{2}_{2}, a^{3}_{1} +l} {|{\alpha ^1 \downarrow a^{1}_{2}, \alpha ^2 \uparrow a^{2}_{2}, \alpha ^3 a^{3}_{1} +l}\rangle } \\ {}&\quad + \sum _{a^{2}_{1} \in {\mathbb {D}}_{1},a^{3}_{2}, a^{1}_{2} \in {\mathbb {D}}_{2}} \eta ^{3} \kappa ^{\alpha ^{1}, \alpha ^{2}, \alpha ^{3}}_{ \downarrow a^{1}_{2}, a^{2}_{1} +l, \uparrow a^{3}_{2}} {|{\alpha ^1 \downarrow a^{1}_{2}, \alpha ^2 a^{2}_{1} +l, \alpha ^3 \uparrow a^{3}_{2}}\rangle } \\&\quad + \sum _{a^{1}_{1} \in {\mathbb {D}}_{1}, a^{2}_{2}, a^{3}_{2} \in {\mathbb {D}}_{2}} \eta ^{4} \kappa ^{\alpha ^{1}, \alpha ^{2}, \alpha ^{3}}_{a^{1}_{1} +l, \uparrow a^{2}_{2}, \downarrow a^{3}_{2}} {|{\alpha ^1 a^{1}_{1} +l, \alpha ^2 \uparrow a^{2}_{2}, \alpha ^3 \downarrow a^{3}_{2}}\rangle } \\ {}&\quad + \sum _{a^{1}_{1} \in {\mathbb {D}}_{1}, a^{2}_{2}, a^{3}_{2} \in {\mathbb {D}}_{2}} \eta ^{5} \kappa ^{\alpha ^{1}, \alpha ^{2}, \alpha ^{3}}_{a^{1}_{1} +l, \downarrow a^{2}_{2}, \uparrow a^{3}_{2}} {|{\alpha ^1 a^{1}_{1} +l, \alpha ^2 \downarrow a^{2}_{2}, \alpha ^3 \uparrow a^{3}_{2}}\rangle }, \end{aligned}$$

where $\alpha ^{i} \in {\mathbb {S}}^{3}$ for $i \in {\mathbb {N}}_3$. After projecting the state by the suitable projector so that in each location $s^1$, $s^2$, and $s^3$ have exactly one particle. Finally, we calculate entanglement with $s^1$ and $s^2$ in spin DoF and between $s^1$ and $s^3$ in spin DoF and OAM DoF respectively. Following the above steps, we have

69 $$\begin{aligned}  {\mathscr {C}}^{2}_{s^1 \mid s^2}&= 4 \left( \kappa ^{s^{1} s^{2}, s^{3}}_{\uparrow a^{1}_{2}, \downarrow a^{2}_{2}, a^{3}_{1} +l} \right) ^2 \left( \kappa ^{s^{1} s^{2}, s^{3}}_{\downarrow a^{1}_{2}, \uparrow a^{2}_{2}, a^{3}_{1} +l} \right) ^2, \\ {\mathscr {C}}^{2}_{s^1 \mid s^3}&=0, \\{\mathscr {C}}^{2}_{s^1 \mid s^2s^3}&= 4 \left( \kappa ^{s^{1} s^{2}, s^{3}}_{\uparrow a^{1}_{2}, \downarrow a^{2}_{2}, a^{3}_{1} +l} \right) ^2 \left( \kappa ^{s^{1} s^{2}, s^{3}}_{\downarrow a^{1}_{2}, \uparrow a^{2}_{2}, a^{3}_{1} +l} \right) ^2. \end{aligned}$$

So, we get monogamy equality as

70 $$\begin{aligned} {\mathscr {C}}^{2}_{s^1 \mid s^2} + {\mathscr {C}}^{2}_{s^1 \mid s^3} = {\mathscr {C}}^{2}_{s^1 \mid s^2s^3}. \end{aligned}$$

*Case 5* All particles are in the different eigenstate of the three different DoFs (say ${|{\uparrow }\rangle }$, ${|{+l}\rangle }$, and ${|{L}\rangle }$ eigenstates of spin, OAM and path DoF respectively). If the particles in the locations ${\mathbb {S}}^3$ are measured in spin, OAM, and path DoF, then we have

71 $$\begin{aligned} {\mathscr {C}}^{2}_{s^1|s^2} = {\mathscr {C}}^{2}_{s^1|s^3}={\mathscr {C}}^{2}_{s^1|s^2s^3}=0. \end{aligned}$$

*Other cases* The above cases consider particles in any of their eigenstates. However, some more cases are possible, where any DoF of any particle at any location might be in a superposition of the eigenstates of that DoF. In such scenarios, one can consider a rotated basis to redefine the eigenstates and the resulting calculations would fall in one of the above cases, owing to the fact that the DoF measurements are localized. For all these cases, we have

72 $$\begin{aligned} {\mathscr {C}}^{2}_{s^1|s^2} = {\mathscr {C}}^{2}_{s^1|s^3}={\mathscr {C}}^{2}_{s^1|s^2s^3}=0. \end{aligned}$$

### Proof of MoE for $p \ge 3$ indistinguishable particles each having *n* DoFs

Suppose there are $p \ge 3$ number of indistinguishable particles, each having *n* DoFs. Recall that, the *k*-th eigenvalue of the *j*th DoF of a particle is represented by ${\mathscr {D}}_{j_{k}} \in {\mathbb {D}}_{j}$ (the set of eigenvalues of the *j*th DoF). As we are considering squared concurrence measure, so we take only two eigenstates of each DoF. For any eigenvalue $\lambda$, we use the notion ${|{\lambda }\rangle }$ for the corresponding eigenstate. In Table 3, we summarize the list of possible combinations to create indistinguishability using three indistinguishable particles, each having three DoFs denoted by *j*, $j^{\prime }$, and $j^{\prime \prime }$, localized in three regions $s^1$, $s^2$, and $s^3$. Calculations for concurrences are done using the method described in “How to calculate the concurrence between any two DoFs of two indistinguishable particles?” section. These cases can be extended for *p* number of indistinguishable particles as shown below.

*Case 1* Entanglement is calculated in the same DoF of all particles. Each particle is in the eigenstate ${|{{\mathscr {D}}}\rangle }_{j_{k}}$ of the *j*th DoF (refer to 1st row of Table 3). Then after calculation, we get ${\mathscr {C}}^{2}_{s^{1} \mid s^{2}} =0$, ${\mathscr {C}}^{2}_{s^{1} \mid s^{3}}= 0$, and ${\mathscr {C}}^{2}_{s^{1} \mid s^{2}s^{3}} = 0$. Similar result holds for *p* indistinguishable particles having the eigenstate ${|{{\mathscr {D}}}\rangle }_{j_{k}}$ of the *j*th DoF.

*Case 2* Entanglement is calculated in the same DoF for all particles (refer to 2nd row of Table 3). For three indistinguishable particles, if two of them are in the eigenstate ${|{{\mathscr {D}}}\rangle }_{j_{k}}$ and one is in the eigenstate ${|{{\mathscr {D}}}\rangle }_{j_{k^{\prime }}}$ where ${|{{\mathscr {D}}}\rangle }_{j_{k^{\prime }}}={|{{\mathscr {D}}}\rangle }^{\perp }_{j_{k}}$, then ${\mathscr {C}}^{2}_{s^{1} \mid s^{2}} \ge 0$, ${\mathscr {C}}^{2}_{s^{1} \mid s^{3}}\ge 0$, and ${\mathscr {C}}^{2}_{s^{1} \mid s^{2}s^{3}} \ge 0$ as shown in “Proof of MoE for three indistinguishable particles each having two DoFs” section. Similar result holds for *p* indistinguishable particles in ${\mathbb {S}}^p$ locations with each particle having *n* DoFs where $(q+r)$ number of particles are in the eigenstate ${|{{\mathscr {D}}}\rangle }_{j_{k}}$ and rest of $(p-q-r)$ number of particles are in the eigenstate ${|{{\mathscr {D}}}\rangle }_{j_{k^{\prime }}}$.

**Table 3:** List of possible combinations to create indistinguishability using three indistinguishable particles localized in three regions $s^1$, $s^2$, and $s^3$, each having three DoFs denoted by *j*, $j^{\prime }$, $j^{\prime \prime }$.

	DoF	Eigenstate	1st particle	2nd particle	3rd particle	Relations	$s^1$	$s^2$	$s^3$	${\mathscr {C}}^{2}_{s^{1} \mid s^{2}}$	${\mathscr {C}}^{2}_{s^{1} \mid s^{3}}$	${\mathscr {C}}^{2}_{s^{1} \mid s^{2}s^{3}}$
							Measures in the DoF					
1	Same	Same	${|{{\mathscr {D}}}\rangle }_{j_{k}}$	${|{{\mathscr {D}}}\rangle }_{j_{k}}$	${|{{\mathscr {D}}}\rangle }_{j_{k}}$	Nil	*j*	*j*	*j*	0	0	0
2	Same	Different	${|{{\mathscr {D}}}\rangle }_{j_{k}}$	${|{{\mathscr {D}}}\rangle }_{j_{k}}$	${|{{\mathscr {D}}}\rangle }_{j_{k^\prime }}$	${\mathscr {D}}_{j_{k}}, {\mathscr {D}}_{j_{k^\prime }} \in {\mathbb {D}}_{j}$, ${|{{\mathscr {D}}}\rangle }_{j_{k^\prime }}={|{{\mathscr {D}}}\rangle }^{\perp }_{j_{k}}$	*j*	*j*	*j*	$\ge 0$	$\ge 0$	$\ge 0$
3	Different	Different	${|{{\mathscr {D}}}\rangle }_{j_{k}}$	${|{{\mathscr {D}}}\rangle }_{j_{k}}$	${|{{\mathscr {D}}}\rangle }_{j^{\prime }_{l}}$	$j \ne j^{\prime }$, ${\mathscr {D}}_{j_{k}} \in {\mathbb {D}}_{j}$, ${\mathscr {D}}_{j^{\prime }_{l}} \in {\mathbb {D}}_{j^{\prime }}$	*j*	*j*	$j^{\prime }$	0	0	0
4	Different	Different	${|{{\mathscr {D}}}\rangle }_{j_{k}}$	${|{{\mathscr {D}}}\rangle }_{j_{k^{\prime }}}$	${|{{\mathscr {D}}}\rangle }_{j^{\prime }_{l}}$	${|{{\mathscr {D}}}\rangle }_{j_{k^\prime }}={|{{\mathscr {D}}}\rangle }^{\perp }_{j_{k}}$, ${\mathscr {D}}_{j^{\prime }_{l}} \in {\mathbb {D}}_{j^{\prime }}$	*j*	*j*	$j^{\prime }$	$\ge 0$	0	$\ge 0$
5	Different	Different	${|{{\mathscr {D}}}\rangle }_{j_{k}}$	${|{{\mathscr {D}}}\rangle }_{j^{\prime \prime }_{h}}$	${|{{\mathscr {D}}}\rangle }_{j^{\prime }_{l}}$	$j \ne j^{\prime } \ne j^{\prime \prime }$, ${\mathscr {D}}_{j^{\prime \prime }_{h}} \in {\mathbb {D}}_{j^{\prime \prime }}$	*j*	$j^{\prime \prime }$	$j^{\prime }$	0	0	0
6	Same	Different	${|{{\mathscr {D}}}\rangle }_{j_{k}}$	${|{{\mathscr {D}}}\rangle }_{j_{k}}$	$\kappa _{j_{k}} {|{{\mathscr {D}}}\rangle }_{j_{k}} + \kappa _{j_{k^{\prime }}} e^{i \phi } {|{{\mathscr {D}}}\rangle }_{j_{k^{\prime }}}$	$\kappa ^{2}_{j_{k}} + \kappa ^{2}_{j_{k^{\prime }}}=1$	*j*	*j*	*j*	$\ge 0$	$\ge 0$	$\ge 0$
7	Same	Different	${|{{\mathscr {D}}}\rangle }_{j_{k}}$	${|{{\mathscr {D}}}\rangle }_{j_{k^{\prime }}}$	$\kappa _{j_{k}} {|{{\mathscr {D}}}\rangle }_{j_{k}} + \kappa _{j_{k^{\prime }}} e^{i \phi } {|{{\mathscr {D}}}\rangle }_{j_{k^{\prime }}}$	${|{{\mathscr {D}}}\rangle }_{j_{k^\prime }}={|{{\mathscr {D}}}\rangle }^{\perp }_{j_{k}}$, $\kappa ^{2}_{j_{k}} + \kappa ^{2}_{j_{k^{\prime }}}=1$	*j*	*j*	*j*	$\ge 0$	$\ge 0$	$\ge 0$
8	Same	Same superposition	$\kappa _{j_{k}} {|{{\mathscr {D}}}\rangle }_{j_{k}} + \kappa _{j_{k^{\prime }}} e^{i \phi } {|{{\mathscr {D}}}\rangle }_{j_{k^{\prime }}}$	$\kappa _{j_{k}} {|{{\mathscr {D}}}\rangle }_{j_{k}} + \kappa _{j_{k^{\prime }}} e^{i \phi } {|{{\mathscr {D}}}\rangle }_{j_{k^{\prime }}}$	$\kappa _{j_{k}} {|{{\mathscr {D}}}\rangle }_{j_{k}} + \kappa _{j_{k^{\prime }}} e^{i \phi } {|{{\mathscr {D}}}\rangle }_{j_{k^{\prime }}}$	$\kappa ^{2}_{j_{k}} + \kappa ^{2}_{j_{k^{\prime }}}=1$	*j*	*j*	*j*	0	0	0
9	Same	Different superposition	$\kappa _{j_{k}} {|{{\mathscr {D}}}\rangle }_{j_{k}} + \kappa _{j_{k^{\prime }}} e^{i \phi _{1}} {|{{\mathscr {D}}}\rangle }_{j_{k^{\prime }}}$, where $\kappa ^{2}_{j_{k}} + \kappa ^{2}_{j_{k^{\prime }}}=1$	$\kappa ^{\prime }_{j_{k}} {|{{\mathscr {D}}}\rangle }_{j_{k}} + \kappa ^{\prime }_{j_{k^{\prime }}} e^{i \phi _{2}} {|{{\mathscr {D}}}\rangle }_{j_{k^{\prime }}}$, where $\kappa ^{\prime ^{ 2}}_{j_{k}} + \kappa ^{\prime ^{2}}_{j_{k^{\prime }}} =1$	$\kappa ^{\prime \prime }_{j_{k}} {|{{\mathscr {D}}}\rangle }_{j_{k}} + \kappa ^{\prime \prime }_{j_{k^{\prime }}} e^{i \phi _{3}} {|{{\mathscr {D}}}\rangle }_{j_{k^{\prime }}}$, where $\kappa ^{\prime \prime ^{2}}_{j_{k}} +\kappa ^{\prime \prime ^{2}}_{j_{k^{\prime }}}=1$	$\phi _1 \ne \phi _2 \ne \phi _3$ $\kappa _{j_{k}} \ne \kappa ^{\prime }_{j_{k}} \ne \kappa ^{\prime \prime }_{j_{k}}$ $\kappa _{j_{k^{\prime }}} \ne \kappa ^{\prime }_{j_{k^{\prime }}} \ne \kappa ^{\prime \prime }_{j_{k^{\prime }}}$	*j*	*j*	*j*	$\ge 0$	$\ge 0$	$\ge 0$
10	Different	Different	${|{{\mathscr {D}}}\rangle }_{j_{k}}$	${|{{\mathscr {D}}}\rangle }_{j_{k}}$	$\kappa _{j^{\prime }_{l}} {|{{\mathscr {D}}}\rangle }_{j^{\prime }_{l}} + \kappa _{j^{\prime }_{l^{\prime }}} e^{i \phi } {|{{\mathscr {D}}}\rangle }_{j^{\prime }_{l^{\prime }}}$	$\kappa ^{2}_{j^{\prime }_{l}} + \kappa ^{2}_{j^{\prime }_{l^{\prime }}}=1$	*j*	*j*	$j^{\prime }$	0	0	0
11	Different	Different	${|{{\mathscr {D}}}\rangle }_{j_{k}}$	${|{{\mathscr {D}}}\rangle }_{j_{k^{\prime }}}$	$\kappa _{j^{\prime }_{l}} {|{{\mathscr {D}}}\rangle }_{j^{\prime }_{l}} + \kappa _{j^{\prime }_{l^{\prime }}} e^{i \phi } {|{{\mathscr {D}}}\rangle }_{j^{\prime }_{l^{\prime }}}$	$\kappa ^{2}_{j^{\prime }_{l}} + \kappa ^{2}_{j^{\prime }_{l^{\prime }}}=1$	*j*	*j*	$j^{\prime }$	$\ge 0$	0	$\ge 0$
12	Different	Different superposition	${|{{\mathscr {D}}}\rangle }_{j_{k}}$	$\kappa _{j_{k}} {|{{\mathscr {D}}}\rangle }_{j_{k}} + \kappa _{j_{k^{\prime }}} e^{i \phi } {|{{\mathscr {D}}}\rangle }_{j_{k^{\prime }}}$, where $\kappa ^{2}_{j_{k}} + \kappa ^{2}_{j_{k^{\prime }}} =1$	$\kappa _{j^{\prime }_{l}} {|{{\mathscr {D}}}\rangle }_{j^{\prime }_{l}} + \kappa _{j^{\prime }_{l^{\prime }}} e^{i \phi } {|{{\mathscr {D}}}\rangle }_{j^{\prime }_{l^{\prime }}}$, where $\kappa ^{2}_{j^{\prime }_{l}} + \kappa ^{2}_{j^{\prime }_{l^{\prime }}}=1$	$j \ne j^{\prime }$	*j*	*j*	$j^{\prime }$	$\ge 0$	$\ge 0$	$\ge 0$
13	Different	Different superposition	$\kappa _{j_{k}} {|{{\mathscr {D}}}\rangle }_{j_{k}} + \kappa _{j_{k^{\prime }}} e^{i \phi } {|{{\mathscr {D}}}\rangle }_{j_{k^{\prime }}}$, where $\kappa ^{2}_{j_{k}} + \kappa ^{2}_{j_{k^{\prime }}}=1$	$\kappa _{j^{\prime \prime }_{h}} {|{{\mathscr {D}}}\rangle }_{j^{\prime \prime }_{h}} + \kappa _{j^{\prime \prime }_{h^{\prime }}} e^{i \phi ^{\prime \prime }} {|{{\mathscr {D}}}\rangle }_{j^{\prime \prime }_{h^{\prime }}}$, where $\kappa ^{2}_{j^{\prime \prime }_{h}} + \kappa ^{2}_{j^{\prime \prime }_{h^{\prime }}} =1$	$\kappa _{j^{\prime }_{l}} {|{{\mathscr {D}}}\rangle }_{j^{\prime }_{l}} + \kappa _{j^{\prime }_{l^{\prime }}} e^{i \phi ^{\prime }} {|{{\mathscr {D}}}\rangle }_{j^{\prime }_{l^{\prime }}}$, where $\kappa ^{2}_{j^{\prime }_{l}} +\kappa ^{2}_{j^{\prime }_{l^{\prime }}}=1$	$j \ne j^{\prime } \ne j^{\prime \prime }$ ${\mathscr {D}}_{j_{k^{\prime }}}, \in {\mathbb {D}}_{j}$, ${\mathscr {D}}_{j^{\prime \prime }_{h^{\prime }}} \in {\mathbb {D}}_{j^{\prime \prime }}$ ${\mathscr {D}}_{j^{\prime }_{l^{\prime }}} \in {\mathbb {D}}_{j^{\prime }}$	*j*	$j^{\prime \prime }$	$j^{\prime }$	0	0	0

Here the second column denotes whether entanglement is calculated in the same DoFs or different DoFs of all particles; the third column denotes whether the eigenstate of the contributing DoFs in entanglement is the same or not or in superposition; the fourth, fifth, and sixth columns describe the eigenstates of the three particles in the corresponding DoFs; the seventh column describes the relations between the eigenstates of the for entanglement. The eighth, ninth, and tenth columns describe the DoF numbers (e.g., *j* means the *j*th DoF) in which the measurements are done in the localized regions $s^1$, $s^2$, and $s^3$ respectively; the rest of the columns represent of the squared concurrences are zero or $\ge 0$.

*Case 3* Entanglement is calculated between two different DoFs. Here, if two particles are in the eigenstate ${|{{\mathscr {D}}}\rangle }_{j_{k}}$ of the *j*th DoF and one particle is in the eigenstate ${|{{\mathscr {D}}}\rangle }_{j^{\prime }_{l}}$ of the $j^{\prime }$th DoF where $j \ne j^{\prime }$ (refer to 3rd row of Table 3), then ${\mathscr {C}}^{2}_{s^{1} \mid s^{2}} = 0$, ${\mathscr {C}}^{2}_{s^{1} \mid s^{3}} = 0$, and ${\mathscr {C}}^{2}_{s^{1} \mid s^{2}s^{3}} = 0$. Similar result holds for *p* indistinguishable particles in ${\mathbb {S}}^p$ locations with each particle having *n* DoFs where $(q+r)$ number of particles are in the eigenstate ${|{{\mathscr {D}}}\rangle }_{j_{k}}$ of the *j*th DoF and rest of $(p-q-r)$ number of particles are in the eigenstate ${|{{\mathscr {D}}}\rangle }_{j^{\prime }_{l}}$ of the $j^{\prime }$th DoF.

*Case 4* Entanglement is calculated between two different DoFs. Here, if two particles are in the eigenstate ${|{{\mathscr {D}}}\rangle }_{j_{k}}$ and ${|{{\mathscr {D}}}\rangle }_{j_{k^{\prime }}}$ of the *j*th DoF respectively and one particle is in the eigenstate ${|{{\mathscr {D}}}\rangle }_{j^{\prime }_{l}}$ of the $j^{\prime }$th DoF where $j \ne j^{\prime }$ and ${|{{\mathscr {D}}}\rangle }_{j_{k^{\prime }}}={|{{\mathscr {D}}}\rangle }^{\perp }_{j_{k}}$ (refer to 4th row of Table 3), then ${\mathscr {C}}^{2}_{s^{1} \mid s^{2}} \ge 0$, ${\mathscr {C}}^{2}_{s^{1} \mid s^{3}} = 0$, and ${\mathscr {C}}^{2}_{s^{1} \mid s^{2}s^{3}} \ge 0$ as shown in “Proof of MoE for three indistinguishable particles each having two DoFs” section. Similar result holds for *p* indistinguishable particles in ${\mathbb {S}}^p$ locations with each particle having *n* DoFs where *q* and *r* number of particles are in the eigenstate ${|{{\mathscr {D}}}\rangle }_{j_{k}}$ and ${|{{\mathscr {D}}}\rangle }_{j_{k^{\prime }}}$ respectively of the *j*th DoF and rest of $(p-q-r)$ number of particles are in the eigenstate ${|{{\mathscr {D}}}\rangle }_{j^{\prime }_{l}}$ of the $j^{\prime }$th DoF.

*Case 5* Entanglement is calculated between three different DoFs of three particles. If three particles are in the eigenstate ${|{{\mathscr {D}}}\rangle }_{j_{k}}$ of the *j*th DoF, ${|{{\mathscr {D}}}\rangle }_{j^{\prime \prime }_{h}}$ of the $j^{\prime \prime }$th DoF, and ${|{{\mathscr {D}}}\rangle }_{j^{\prime }_{l}}$ of the $j^{\prime }$th DoF where $j \ne j^{\prime } \ne j^{\prime \prime }$, then ${\mathscr {C}}^{2}_{s^{1} \mid s^{2}} = 0$, ${\mathscr {C}}^{2}_{s^{1} \mid s^{3}} = 0$, and ${\mathscr {C}}^{2}_{s^{1} \mid s^{2}s^{3}} = 0$. Similar result holds for *p* indistinguishable particles in ${\mathbb {S}}^p$ locations with each particle having *n* DoFs where *q* number of particles are in the the eigenstate ${|{{\mathscr {D}}}\rangle }_{j_{k}}$ of *j*th DoF, *r* number of particles are in the eigenstate ${|{{\mathscr {D}}}\rangle }_{j^{\prime \prime }_{h}}$ of $j^{\prime \prime }$th DoF and rest of $(p-q-r)$ number of particles are in the eigenstate ${|{{\mathscr {D}}}\rangle }_{j^{\prime }_{l}}$ of the $j^{\prime }$th DoF (refer to 5th row of Table 3).

*Case 6* Entanglement is calculated in the same DoF of all particles. If two particles are in ${|{{\mathscr {D}}}\rangle }_{j_{k}}$ and one particle is in the superpositions of its eigenstate, i.e., $\kappa _{j_{k}} {|{{\mathscr {D}}}\rangle }_{j_{k}} + \kappa _{j_{k^{\prime }}} e^{i \phi } {|{{\mathscr {D}}}\rangle }_{j_{k^{\prime }}}$ where $\kappa ^{2}_{j_{k}} + \kappa ^{2}_{j_{k^{\prime }}}=1$, then ${\mathscr {C}}^{2}_{s^{1} \mid s^{2}} \ge 0$, ${\mathscr {C}}^{2}_{s^{1} \mid s^{3}} \ge 0$, and ${\mathscr {C}}^{2}_{s^{1} \mid s^{2}s^{3}} \ge 0$. The calculations are similar to case 2. Similar result holds for *p* indistinguishable particles in ${\mathbb {S}}^p$ locations with each particle having *n* DoFs where $(q+r)$ particles are in ${|{{\mathscr {D}}}\rangle }_{j_{k}}$ and rest of $(p-q-r)$ particles are in the superpositions of its eigenstate, i.e., $\kappa _{j_{k}} {|{{\mathscr {D}}}\rangle }_{j_{k}} + \kappa _{j_{k^{\prime }}} e^{i \phi } {|{{\mathscr {D}}}\rangle }_{j_{k^{\prime }}}$ (refer to 6th row of Table 3).

*Case 7* Entanglement is calculated in the same DoF of all particles. If two particles are in the eigenstate ${|{{\mathscr {D}}}\rangle }_{j_{k}}$ and ${|{{\mathscr {D}}}\rangle }_{j_{k^{\prime }}}$ and one particle is in superpositions of its eigenstate, i.e., $\kappa _{j_{k}} {|{{\mathscr {D}}}\rangle }_{j_{k}} + \kappa _{j_{k^{\prime }}} e^{i \phi } {|{{\mathscr {D}}}\rangle }_{j_{k^{\prime }}}$ where $\kappa ^{2}_{j_{k}} + \kappa ^{2}_{j_{k^{\prime }}}=1$ of the *j*th DoF, then ${\mathscr {C}}^{2}_{s^{1} \mid s^{2}} \ge 0$, ${\mathscr {C}}^{2}_{s^{1} \mid s^{3}} \ge 0$, and ${\mathscr {C}}^{2}_{s^{1} \mid s^{2}s^{3}} \ge 0$. The calculations are similar to case 2. A similar result holds for *p* indistinguishable particles in ${\mathbb {S}}^p$ locations with each particle having *n* DoFs where *q* number of particles are in ${|{{\mathscr {D}}}\rangle }_{j_{k}}$, *r* number of particles are in ${|{{\mathscr {D}}}\rangle }_{j_{k^{\prime }}}$ and rest of $(p-q-r)$ number of particles are in superpositions of its eigenstate, i.e., $\kappa _{j_{k}} {|{{\mathscr {D}}}\rangle }_{j_{k}} + \kappa _{j_{k^{\prime }}} e^{i \phi } {|{{\mathscr {D}}}\rangle }_{j_{k^{\prime }}}$ where $\kappa ^{2}_{j_{k}} + \kappa ^{2}_{j_{k^{\prime }}}=1$ (refer to 7th row of Table 3).

*Case 8* Entanglement is calculated in the same DoF of all particles. Each particle are in the superpositions of its eigenstate, i.e., $\kappa _{j_{k}} {|{{\mathscr {D}}}\rangle }_{j_{k}} + \kappa _{j_{k^{\prime }}} e^{i \phi } {|{{\mathscr {D}}}\rangle }_{j_{k^{\prime }}}$ where $\kappa ^{2}_{j_{k}} + \kappa ^{2}_{j_{k^{\prime }}}=1$. Now calculations show that ${\mathscr {C}}^{2}_{s^{1} \mid s^{2}} = 0$, ${\mathscr {C}}^{2}_{s^{1} \mid s^{3}} = 0$, and ${\mathscr {C}}^{2}_{s^{1} \mid s^{2}s^{3}} = 0$. This case is similar to case 1 if we take a rotated basis to redefine the eigenstates as $\lbrace {|{{\tilde{\mathscr {D}}}}\rangle }_{j_{k}}, {|{{\tilde{\mathscr {D}}}}\rangle }^{\perp }_{j_{k}} \rbrace$ where ${|{{\tilde{\mathscr {D}}}}\rangle }_{j_{k}} = \kappa _{j_{k}} {|{{\mathscr {D}}}\rangle }_{j_{k}} + \kappa _{j_{k^{\prime }}} e^{i \phi } {|{{\mathscr {D}}}\rangle }_{j_{k^{\prime }}}$ (refer to 8th row of Table 3).

*Case 9* Entanglement is calculated in the same DoF of all particles. Each particles are in different superpositions of its eigenstate, i.e., three particles are in the eigenstates $\kappa _{j_{k}} {|{{\mathscr {D}}}\rangle }_{j_{k}} + \kappa _{j_{k^{\prime }}} e^{i \phi _{1}} {|{{\mathscr {D}}}\rangle }_{j_{k^{\prime }}}$, $\kappa ^{\prime }_{j_{k}} {|{{\mathscr {D}}}\rangle }_{j_{k}} + \kappa ^{\prime }_{j_{k^{\prime }}} e^{i \phi _{2}} {|{{\mathscr {D}}}\rangle }_{j_{k^{\prime }}}$, and $\kappa ^{\prime \prime }_{j_{k}} {|{{\mathscr {D}}}\rangle }_{j_{k}} + \kappa ^{\prime \prime }_{j_{k^{\prime }}} e^{i \phi _{3}} {|{{\mathscr {D}}}\rangle }_{j_{k^{\prime }}}$ of the *j*th DoF where $\kappa ^{2}_{j_{k}} + \kappa ^{2}_{j_{k^{\prime }}}=1$ , $\kappa ^{2}_{j^{\prime \prime }_{h}} + \kappa ^{2}_{j^{\prime \prime }_{h^{\prime }}} =1$, $\kappa ^{2}_{j^{\prime }_{l}} +\kappa ^{2}_{j^{\prime }_{l^{\prime }}}=1$, $\phi _1 \ne \phi _2 \ne \phi _3$, $\kappa _{j_{k}} \ne \kappa ^{\prime }_{j_{k}} \ne \kappa ^{\prime \prime }_{j_{k}}$, and $\kappa _{j_{k^{\prime }}} \ne \kappa ^{\prime }_{j_{k^{\prime }}} \ne \kappa ^{\prime \prime }_{j_{k^{\prime }}}$. Now calculations show that ${\mathscr {C}}^{2}_{s^{1} \mid s^{2}} \ge 0$, ${\mathscr {C}}^{2}_{s^{1} \mid s^{3}} \ge 0$, and ${\mathscr {C}}^{2}_{s^{1} \mid s^{2}s^{3}} \ge 0$. The calculations are similar to case 8. Similar result holds for *p* indistinguishable particles in ${\mathbb {S}}^p$ locations with each particle having *n* DoFs where *q* number particles are in $\kappa _{j_{k}} {|{{\mathscr {D}}}\rangle }_{j_{k}} + \kappa _{j_{k^{\prime }}} e^{i \phi _{1}} {|{{\mathscr {D}}}\rangle }_{j_{k^{\prime }}}$ eigenstate, *r* number particles are in $\kappa ^{\prime }_{j_{k}} {|{{\mathscr {D}}}\rangle }_{j_{k}} + \kappa ^{\prime }_{j_{k^{\prime }}} e^{i \phi _{2}} {|{{\mathscr {D}}}\rangle }_{j_{k^{\prime }}}$ eigenstate and $(p-q-r)$ number of particles are in $\kappa ^{\prime \prime }_{j_{k}} {|{{\mathscr {D}}}\rangle }_{j_{k}} + \kappa ^{\prime \prime }_{j_{k^{\prime }}} e^{i \phi _{3}} {|{{\mathscr {D}}}\rangle }_{j_{k^{\prime }}}$ eigenstate (refer to 9th row of Table 3).

*Case 10* Here entanglement is calculated among two different DoFs where two particles are in ${|{{\mathscr {D}}}\rangle }_{j_{k}}$ eigenstate of the *j*th DoF and one particle is in $\kappa _{j^{\prime }_{l}} {|{{\mathscr {D}}}\rangle }_{j^{\prime }_{l}} + \kappa _{j^{\prime }_{l^{\prime }}} e^{i \phi } {|{{\mathscr {D}}}\rangle }_{j^{\prime }_{l^{\prime }}}$ eigenstate in the $j^{\prime }$th DoF. Here $j, j^{\prime } \in {\mathbb {N}}_{n}$ and $\kappa ^{2}_{j^{\prime }_{l}} + \kappa ^{2}_{j^{\prime }_{l^{\prime }}}=1$. Now calculations show ${\mathscr {C}}^{2}_{s^{1} \mid s^{2}} = 0$, ${\mathscr {C}}^{2}_{s^{1} \mid s^{3}} = 0$, and ${\mathscr {C}}^{2}_{s^{1} \mid s^{2}s^{3}} = 0$. This calculation is easier if we take a rotated basis as shown in case 8. Similar result holds for *p* indistinguishable particles in ${\mathbb {S}}^p$ locations with each particle having *n* DoFs where $(q+r)$ number of particles are in ${|{{\mathscr {D}}}\rangle }_{j_{k}}$ eigenstate of the *j*th DoF and rest of $(p-q-r)$ number of particles are in $\kappa _{j^{\prime }_{l}} {|{{\mathscr {D}}}\rangle }_{j^{\prime }_{l}} + \kappa _{j^{\prime }_{l^{\prime }}} e^{i \phi } {|{{\mathscr {D}}}\rangle }_{j^{\prime }_{l^{\prime }}}$ eigenstate in the $j^{\prime }$th DoF (refer to 10th row of Table 3).

*Case 11* Here entanglement is calculated among two different DoFs where two particles are in ${|{{\mathscr {D}}}\rangle }_{j_{k}}$ and ${|{{\mathscr {D}}}\rangle }_{j_{k^{\prime }}}$ eigenstate of the *j*th DoF and one particle is in $\kappa _{j^{\prime }_{l}} {|{{\mathscr {D}}}\rangle }_{j^{\prime }_{l}} + \kappa _{j^{\prime }_{l^{\prime }}} e^{i \phi } {|{{\mathscr {D}}}\rangle }_{j^{\prime }_{l^{\prime }}}$ eigenstate in the $j^{\prime }$th DoF. Here $j, j^{\prime } \in {\mathbb {N}}_{n}$ and $\kappa ^{2}_{j^{\prime }_{l}} + \kappa ^{2}_{j^{\prime }_{l^{\prime }}}=1$. Now calculations show that ${\mathscr {C}}^{2}_{s^{1} \mid s^{2}} \ge 0$, ${\mathscr {C}}^{2}_{s^{1} \mid s^{3}} = 0$, and ${\mathscr {C}}^{2}_{s^{1} \mid s^{2}s^{3}} \ge 0$. If we consider a rotated basis in $j^{\prime }$ DoF as $\lbrace {|{{\tilde{\mathscr {D}}}}\rangle }_{j^{\prime }_{l}}, {|{{\tilde{\mathscr {D}}}}\rangle }^{\perp }_{j^{\prime }_{l}} \rbrace$ where ${|{{\tilde{\mathscr {D}}}}\rangle }_{j^{\prime }_{l}} =\kappa _{j^{\prime }_{l}} {|{{\mathscr {D}}}\rangle }_{j^{\prime }_{l}} + \kappa _{j^{\prime }_{l^{\prime }}} e^{i \phi } {|{{\mathscr {D}}}\rangle }_{j^{\prime }_{l^{\prime }}}$, then the calculations is similar as case 3. Similar result holds for *p* indistinguishable particles in ${\mathbb {S}}^p$ locations with each particle having *n* DoFs where *q* and *r* number of particles are in ${|{{\mathscr {D}}}\rangle }_{j_{k}}$ and ${|{{\mathscr {D}}}\rangle }_{j_{k^{\prime }}}$ eigenstate respectively of the *j*th DoF and rest of $(p-q-r)$ number of particles are in $\kappa _{j^{\prime }_{l}} {|{{\mathscr {D}}}\rangle }_{j^{\prime }_{l}} + \kappa _{j^{\prime }_{l^{\prime }}} e^{i \phi } {|{{\mathscr {D}}}\rangle }_{j^{\prime }_{l^{\prime }}}$ eigenstate in $j^{\prime }$th DoF (refer to 11th row of Table 3).

*Case 12* Here entanglement is calculated among two different DoFs two particles are in the ${|{{\mathscr {D}}}\rangle }_{j_{k}}$ eigenstate and $\kappa _{j_{k}} {|{{\mathscr {D}}}\rangle }_{j_{k}} + \kappa _{j_{k^{\prime }}} e^{i \phi } {|{{\mathscr {D}}}\rangle }_{j_{k^{\prime }}}$ eigenstate in the *j*th DoF, one particle is in the superposition, i.e., $\kappa _{j^{\prime }_{l}} {|{{\mathscr {D}}_{j^{\prime }_{l}}}\rangle } + \kappa _{j^{\prime }_{l^{\prime }}} e^{i \phi } {|{{\mathscr {D}}}\rangle }_{j^{\prime }_{l^{\prime }}}$ eigenstate in the $j^{\prime }$th DoF. Now calculations show ${\mathscr {C}}^{2}_{s^{1} \mid s^{2}} \ge 0$, ${\mathscr {C}}^{2}_{s^{1} \mid s^{3}} = 0$, and ${\mathscr {C}}^{2}_{s^{1} \mid s^{2}s^{3}} \ge 0$. Using an appropriate rotated basis of the *j*th and $j^{\prime }$th DoF, the calculations are similar to the previous case. Similar result holds for *p* indistinguishable particles in ${\mathbb {S}}^p$ locations with each particle having *n* DoFs where *q* number of particles are in ${|{{\mathscr {D}}}\rangle }_{j_{k}}$ eigenstate of the *j*th DoF, *r* number of particles are in the superposition, i.e., $\kappa _{j_{k}} {|{{\mathscr {D}}}\rangle }_{j_{k}} + \kappa _{j_{k^{\prime }}} e^{i \phi } {|{{\mathscr {D}}}\rangle }_{j_{k^{\prime }}}$ eigenstate in the *j*th DoF, and rest of $(p-q-r)$ number of particles are in the superposition, i.e., $\kappa _{j^{\prime }_{l}} {|{{\mathscr {D}}_{j^{\prime }_{l}}}\rangle } + \kappa _{j^{\prime }_{l^{\prime }}} e^{i \phi } {|{{\mathscr {D}}}\rangle }_{j^{\prime }_{l^{\prime }}}$ eigenstate in the $j^{\prime }$th DoF (refer to 12th row of Table 3).

*Case 13* Entanglement is calculated between three different DoFs. Here, three particles are in the superpositions of its eigenstate, i.e., $\kappa _{j_{k}} {|{{\mathscr {D}}}\rangle }_{j_{k}} + \kappa _{j_{k^{\prime }}} e^{i \phi } {|{{\mathscr {D}}}\rangle }_{j_{k^{\prime }}}$ where $\kappa ^{2}_{j_{k}} + \kappa ^{2}_{j_{k^{\prime }}}=1$ of the *j*th DoF; $\kappa _{j^{\prime \prime }_{h}} {|{{\mathscr {D}}}\rangle }_{j^{\prime \prime }_{h}} + \kappa _{j^{\prime \prime }_{h^{\prime }}} e^{i \phi ^{\prime \prime }} {|{{\mathscr {D}}}\rangle }_{j^{\prime \prime }_{h^{\prime }}}$ where $\kappa ^{2}_{j^{\prime \prime }_{h}} + \kappa ^{2}_{j^{\prime \prime }_{h^{\prime }}} =1$ of the $j^{\prime \prime }$th DoF; and $\kappa _{j^{\prime }_{l}} {|{{\mathscr {D}}}\rangle }_{j^{\prime }_{l}} + \kappa _{j^{\prime }_{l^{\prime }}} e^{i \phi ^{\prime }} {|{{\mathscr {D}}}\rangle }_{j^{\prime }_{l^{\prime }}}$ where $\kappa ^{2}_{j^{\prime }_{l}} +\kappa ^{2}_{j^{\prime }_{l^{\prime }}}=1$ of the $j^{\prime }$th DoF where $j \ne j^{\prime } \ne j^{\prime \prime }$. Using an appropriate rotated basis of the *j*th, $j^{\prime }$th, and $j^{\prime \prime }$th DoF, the calculations are similar as shown in case 5. Now calculations show ${\mathscr {C}}^{2}_{s^{1} \mid s^{2}} = 0$, ${\mathscr {C}}^{2}_{s^{1} \mid s^{3}} = 0$, and ${\mathscr {C}}^{2}_{s^{1} \mid s^{2}s^{3}} = 0$. Similar result holds for *p* indistinguishable particles in ${\mathbb {S}}^p$ locations with each particle having *n* DoFs where *q* number of particles are in superpositions of its eigenstate, i.e., $\kappa _{j_{k}} {|{{\mathscr {D}}}\rangle }_{j_{k}} + \kappa _{j_{k^{\prime }}} e^{i \phi } {|{{\mathscr {D}}}\rangle }_{j_{k^{\prime }}}$ of the *j*th DoF, Here, *r* number of particles are in superpositions of its eigenstate, i.e., $\kappa _{j^{\prime \prime }_{h}} {|{{\mathscr {D}}}\rangle }_{j^{\prime \prime }_{h}} + \kappa _{j^{\prime \prime }_{h^{\prime }}} e^{i \phi ^{\prime \prime }} {|{{\mathscr {D}}}\rangle }_{j^{\prime \prime }_{h^{\prime }}}$ of $j^{\prime \prime }$th DoF and rest of $(p-q-r)$ number of particles are in superpositions of its eigenstate, i.e., $\kappa _{j^{\prime }_{l}} {|{{\mathscr {D}}}\rangle }_{j^{\prime }_{l}} + \kappa _{j^{\prime }_{l^{\prime }}} e^{i \phi ^{\prime }} {|{{\mathscr {D}}}\rangle }_{j^{\prime }_{l^{\prime }}}$ of $j^{\prime }$th DoF (refer to 13th row of Table 3).

One may think that there might be more cases. Upon careful inspection, it can be concluded that all those cases are equivalent to any of the above-mentioned cases.

### Proof of MoE indistinguishable particles for mixed states

In this section, we generalize the relation for monogamy of entanglement of indistinguishable particles for mixed states. We have proved in Corollary 1.1 the main text that for all pure states $\rho _{\alpha _{i} \beta _{j} \gamma _{k}}$

73 $$\begin{aligned} {\mathscr {C}}^{2}_{\alpha _{i} \mid \beta _{j}} \left( \rho _{\alpha _{i} \beta _{j} }\right) +{\mathscr {C}}^{2}_{\alpha _{i} \mid \gamma _{k}}\left( \rho _{\alpha _{i} \mid \gamma _{k} } \right) = {\mathscr {C}}^{2}_{\alpha _{i} \mid \beta _{j} \gamma _{k}} \left( \rho _{\alpha _{i} \beta _{j} \gamma _{k}}\right) . \end{aligned}$$

But this relation is not valid for mixed states as the right-hand side is not defined for mixed states. Since all mixed states are convex combinations some pure states, we can write $\rho _{\alpha _{i} \beta _{j} \gamma _{k}}$ as a convex combination of pure states, as

74 $$\begin{aligned} \rho _{\alpha _{i} \beta _{j} \gamma _{k}}= \sum _{m} \text {Pr}_{m} {|{\psi _{m}}\rangle }_{\alpha _{i} \beta _{j} \gamma _{k}} {\langle {\psi _{m}}|}_{\alpha _{i} \beta _{j} \gamma _{k}}, \end{aligned}$$

where $\Pr _{m}$ denotes the probability of ${|{\psi _{m}}\rangle }_{\alpha _{i} \beta _{j} \gamma _{k}}$. For each *m*, we can write from Eq. (73) as

75 $$\begin{aligned} {\mathscr {C}}^{2}_{\alpha _{i} \mid \beta _{j}} \left( {|{\psi _{m}}\rangle }_{\alpha _{i} \beta _{j}} {\langle {\psi _{m}}|}_{\alpha _{i} \beta _{j}} \right) + {\mathscr {C}}^{2}_{\alpha _{i} \mid \gamma _{k}} \left( {|{\psi _{m}}\rangle }_{\alpha _{i} \gamma _{k}} {\langle {\psi _{m}}|}_{\alpha _{i} \gamma _{k}} \right) = {\mathscr {C}}^{2}_{\alpha _{i} \mid \beta _{j}\gamma _{k}} \left( {|{\psi _{m}}\rangle }_{\alpha _{i} \beta _{j}\gamma _{k}} {\langle {\psi _{m}}|}_{\alpha _{i} \beta _{j}\gamma _{k}} \right) . \end{aligned}$$

Multiplying both sides with $\text {Pr}_{m}$, we get

76 $$\begin{aligned} \text {Pr}_{m} {\mathscr {C}}^{2}_{\alpha _{i} \mid \beta _{j}} \left( {|{\psi _{m}}\rangle }_{\alpha _{i} \beta _{j}} {\langle {\psi _{m}}|}_{\alpha _{i} \beta _{j}} \right) + \text {Pr}_{m} {\mathscr {C}}^{2}_{\alpha _{i} \mid \gamma _{k}} \left( {|{\psi _{m}}\rangle }_{\alpha _{i} \gamma _{k}} {\langle {\psi _{m}}|}_{\alpha _{i} \gamma _{k}} \right) = \text {Pr}_{m} {\mathscr {C}}^{2}_{\alpha _{i} \mid \beta _{j}\gamma _{k}} \left( {|{\psi _{m}}\rangle }_{\alpha _{i} \beta _{j}\gamma _{k}} {\langle {\psi _{m}}|}_{\alpha _{i} \beta _{j}\gamma _{k}} \right) . \end{aligned}$$

Summing up for all the pure constituents,

77 $$\begin{aligned} \sum _{m} \text {Pr}_{m} {\mathscr {C}}^{2}_{\alpha _{i} \mid \beta _{j}} \left( {|{\psi _{m}}\rangle }_{\alpha _{i} \beta _{j}} {\langle {\psi _{m}}|}_{\alpha _{i} \beta _{j}} \right) + \sum _{m}\text {Pr}_{m} {\mathscr {C}}^{2}_{\alpha _{i} \mid \gamma _{k}} \left( {|{\psi _{m}}\rangle }_{\alpha _{i} \gamma _{k}} {\langle {\psi _{m}}|}_{\alpha _{i} \gamma _{k}} \right) = \sum _{m}\text {Pr}_{m} {\mathscr {C}}^{2}_{\alpha _{i} \mid \beta _{j}\gamma _{k}} \left( {|{\psi _{m}}\rangle }_{\alpha _{i} \beta _{j}\gamma _{k}} {\langle {\psi _{m}}|}_{\alpha _{i} \beta _{j}\gamma _{k}} \right) . \end{aligned}$$

Now consider the decomposition, say $\left\{ \left( \text {Pr}^{*}_{m}, {|{\psi _{m}}\rangle }^{*}_{\alpha _{i} \beta _{j} \gamma _{k}} \right) \right\}$, that minimizes the right hand side of Eq. (77) and denote it by

78 $$\begin{aligned} \left( {\mathscr {C}}^{2}_{\alpha _{i} \mid \beta _{j} \gamma _{k}}\right) ^{\text {min}}:= \min _{ \left\{ \left( \text {Pr}_{m}, {|{\psi _{m}}\rangle }_{\alpha _{i} \beta _{j} \gamma _{k}} \right) \right\} } \sum _{m}\text {Pr}_{m} {\mathscr {C}}^{2}_{\alpha _{i} \mid \beta _{j}\gamma _{k}} \left( {|{\psi _{m}}\rangle }_{\alpha _{i} \beta _{j}\gamma _{k}} {\langle {\psi _{m}}|}_{\alpha _{i} \beta _{j}\gamma _{k}} \right) . \end{aligned}$$

Now expressing $\rho _{\alpha _{i} \beta _{j} \gamma _{k}}$ by minimizing the above decomposition as in Eq. (78), we have^40^

79 $$\begin{aligned} &{\mathscr {C}}^{2}_{\alpha _{i} \mid \beta _{j}} \left( \rho _{\alpha _{i} \beta _{j} }\right) +{\mathscr {C}}^{2}_{\alpha _{i} \mid \gamma _{k}}\left( \rho _{\alpha _{i} \mid \gamma _{k} } \right) \\&= {\mathscr {C}}^{2}_{\alpha _{i} \mid \beta _{j}} \left( \sum _{m} \text {Pr}^{*}_{m}{|{\psi _{m}}\rangle }^{*}_{\alpha _{i} \beta _{j}} {\langle {\psi _{m}}|}^{*}_{\alpha _{i} \beta _{j}} \right) + {\mathscr {C}}^{2}_{\alpha _{i} \mid \gamma _{k}} \left( \sum _{m} \text {Pr}^{*}_{m}{|{\psi _{m}}\rangle }^{*}_{\alpha _{i} \gamma _{k}} {\langle {\psi _{m}}|}^{*}_{\alpha _{i} \gamma _{k}} \right) \\&\le \sum _{m} \text {Pr}^{*}_{m} {\mathscr {C}}^{2}_{\alpha _{i} \mid \beta _{j}} \left( {|{\psi _{m}}\rangle }^{*}_{\alpha _{i} \beta _{j}} {\langle {\psi _{m}}|}^{*}_{\alpha _{i} \beta _{j}} \right) + \sum _{m}\text {Pr}^{*}_{m} {\mathscr {C}}^{2}_{\alpha _{i} \mid \gamma _{k}} \left( {|{\psi _{m}}\rangle }^{*}_{\alpha _{i} \gamma _{k}} {\langle {\psi _{m}}|}^{*}_{\alpha _{i} \gamma _{k}} \right) \hspace{1cm} \text {(by the convexity of }{\mathscr {C}}^{2}\text { )}\\&= \sum _{m} \text {Pr}^{*}_{m} \left\{ {\mathscr {C}}^{2}_{\alpha _{i} \mid \beta _{j}} \left( {|{\psi _{m}}\rangle }^{*}_{\alpha _{i} \beta _{j}} {\langle {\psi _{m}}|}^{*}_{\alpha _{i} \beta _{j}} \right) + {\mathscr {C}}^{2}_{\alpha _{i} \mid \gamma _{k}} \left( {|{\psi _{m}}\rangle }^{*}_{\alpha _{i} \gamma _{k}} {\langle {\psi _{m}}|}^{*}_{\alpha _{i} \gamma _{k}} \right) \right\} \\&= \sum _{m}\text {Pr}^{*}_{m} {\mathscr {C}}^{2}_{\alpha _{i} \mid \beta _{j}\gamma _{k}} \left( {|{\psi _{m}}\rangle }^{*}_{\alpha _{i} \beta _{j}\gamma _{k}} {\langle {\psi _{m}}|}^{*}_{\alpha _{i} \beta _{j}\gamma _{k}} \right) \hspace{1cm} \left( \text {by Eq.}~(75) \right) \\&= \left( {\mathscr {C}}^{2}_{\alpha _{i} \mid \beta _{j}\gamma _{k}}\right) ^{\text {min}} \hspace{1cm} \left( \text {from Eq.}~(78) \right) . \end{aligned}$$

Thus we have for mixed states

80 $$\begin{aligned} {\mathscr {C}}^{2}_{\alpha _{i} \mid \beta _{j}} \left( \rho _{\alpha _{i} \beta _{j} }\right) +{\mathscr {C}}^{2}_{\alpha _{i} \mid \gamma _{k}}\left( \rho _{\alpha _{i} \mid \gamma _{k} } \right) \le {\mathscr {C}}^{2}_{\alpha _{i} \mid \beta _{j} \gamma _{k}} \left( \rho _{\alpha _{i} \beta _{j} \gamma _{k}}\right) . \end{aligned}$$

### Supplementary Information

Supplementary Information.

## Data Availability

All data generated or analysed during this study are included in this published article [and its Supplementary Information files].
