# Supplementary material for: Entanglement monogamy in indistinguishable particle systems
Source: Sci Rep. 2023 Dec 11;13:21972. doi: 10.1038/s41598-023-46515-z (PMC10713569; doi:10.1038/s41598-023-46515-z)
Supplement: Supplementary file 1 — Supplementary Information. [file 41598_2023_46515_MOESM1_ESM.pdf]

Supplemental Material
Entanglement Monogamy in Indistinguishable Particle Systems

Soumya Das[1,*], **Goutam Paul**[1,+], **and Ritabrata Sengupta**[2,+]

[1]Cryptology and Security Research Unit, R. C. Bose Centre for Cryptology and Security, Indian Statistical Institute, Kolkata 700108, India
[2]Department of Mathematical Sciences, Indian Institute of Science Education and Research Berhampur, Transit Campus, Government ITI, Berhampur 760010, Odisha, India
[*]soumya06.das@gmail.com
[+]goutam.paul@isical.ac.in (*Corresponding Author*)
[!]rb@iiserbpr.ac.in

1 Can we represent different DoFs as different particles?

In this section, we discuss: if we represent different DoFs as different particles, can we get the same result? We answer this question with the following example.

Suppose a particle A made of n DoFs each of dimension d, then the total dimension of A is $D_A = d^n$. Now consider another particle B of dimension $D_B = d^n$. For both the particles, the cardinality of the dimension is the same, $\mid D_A \mid = \mid D_B \mid$. So are they really equivalent?

If we just compare the cardinality of the dimensions, then they are the same. However, if we consider the Hilbert Space structure, then they are completely different. The Hilbert space structure of the particle A is $(\underbrace{\mathscr{C}_d \otimes \mathscr{C}_d \otimes \ldots \otimes \mathscr{C}_d}_{n\text{-times}})$ whereas the Hilbert space structure of the particle B is $\mathscr{C}_{d^n}$. Thus the two particles A and B may have the same dimension but physically they are not the same.

Now take another situation where on one side, there is a particle A made of n DoFs each of dimension d and on another side, there is n number of particles each having a single DoF with dimension d. In this case, both the dimension and the Hilbert Space structure of both sides are the same. Now are these two cases equivalent?

The answer is negative from the operational point of view. To calculate monogamy, we need to trace-out a specific DoF from some specific particle. Suppose there are two particles A and B each having n DoFs are entangled in all their DoFs. If we want to trace-out i-th DoF of particle A, we get the reduced state of the joint system where are are two particles one having n number of DoFs (for particle B) and another particle having $(n-1)$ number of DoF (for particle A). If we represent all the DoFs of particles A and B as different particles then it is not possible. This situation becomes more complicated when particles become indistinguishable. Thus we need to have a separate representation of indistinguishable particles having multiple DoFs.

2 How to create two indistinguishable particles by spatial overlap

In Fig. 1, it is shown schematically how two particles with opposite spins are made indistinguishable. Due to unitarity, it is actually impossible to deterministically place two particles into the same spot without the aid of interactions. The particle exchange method as proposed in[1,2] is such an interaction that creates wave-function overlap without physically placing two particles in the same location. Using linear optics, this operation can be done by beam splitters and phase shifters as shown in [1, Fig. 1]. The unitary relation for the beam splitter is given by

$$\frac{1}{\sqrt{2}}\begin{pmatrix} 1 & 1 \\ 1 & -1 \end{pmatrix}. \tag{1}$$

In Fig. 1 (a), there are two particles are in two regions s^1 and s^2 having $|\uparrow\rangle$ and $|\downarrow\rangle$ eigenstates in spin degrees of freedom. When s^1 and s^2 are non-overlapping, these two particles can be distinguished by their individual spatial labels, they remain separable and can be represented as

$$|\psi\rangle_{dis} = |\uparrow\rangle_{s^1} \otimes |\downarrow\rangle_{s^2}. \tag{2}$$

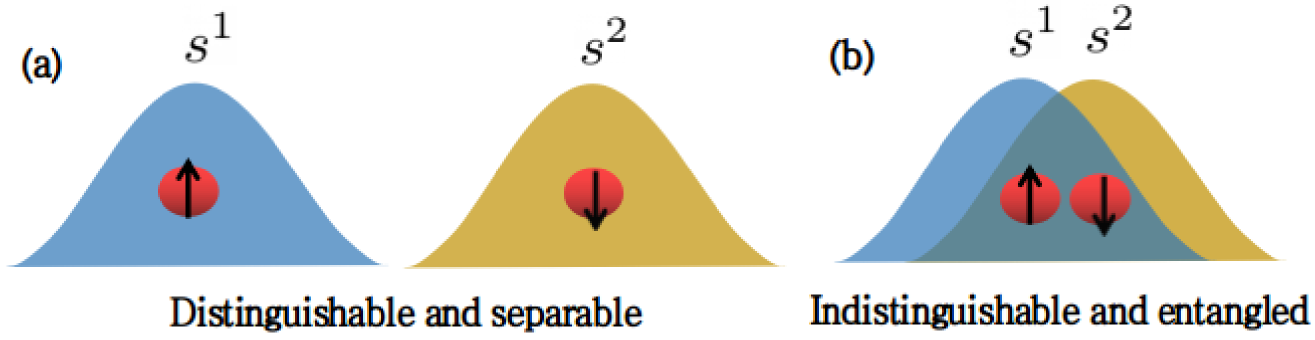

Figure 1. Two particles are in two regions s^1 and s^2 having $|\uparrow\rangle$ and $|\downarrow\rangle$ eigenstates in spin degrees of freedom. (a) When s^1 and s^2 are non-overlapping, these two particles can be distinguished by their individual spatial labels, they remain separable and can be represented as $|\uparrow\rangle_{s^1} \otimes |\downarrow\rangle_{s^2}$. (b) When there is an overlap between s^1 and s^2, they cannot be identified by their individual spatial labels, they become entangled and are represented as $|s^1 \uparrow, s^2 \downarrow\rangle$.

When there is an overlap between s^1 and s^2 as shown in In Fig. 1 (b), they cannot be identified by their individual spatial labels, they become entangled and are represented as

$$|\psi\rangle_{indis} = |s^1 \uparrow, s^2 \downarrow\rangle \,. \tag{3}$$

3 Are the measurements for distinguishable and indistinguishable particles the same?

It is a common question that whether the measurements for distinguishable and indistinguishable particles are the same. if so then how one can get something that is possible for distinguishable particles and impossible for indistinguishable particles (or vice-versa)? The reason for this problem is when monogamy arises due to entanglement between particles the only way to measure that entanglement is via detectors, i.e., particles whether distinguishable or indistinguishable must be detected somewhere. Then from the measurement result, we can calculate entanglement.

For distinguishable particles, the relation between particles and the detectors is unambiguous because the particles can be addressed individually throughout the experiment. Thus the particles intended for a particular detector are detected on that detector. But for indistinguishable particles, this relation is ambiguous as during the experiment, the particles cannot be addressed individually. Thus when they are detected in a detector, we cannot say about the source of that particle. However, the process of measurement for both distinguishable and indistinguishable particles is the same.

Then the next question arises using the detectors and quantum state tomography, we can always reconstruct the corresponding quantum state irrespective of the particles being distinguishable or indistinguishable. Suppose for three detectors A, B, and C, the measurement results can be represented using some density matrix ρ_{ABC}. Now the question is whether every physically allowed ρ_{ABC} can be implemented using distinguishable or indistinguishable particles.

The answer is no. We cannot implement any physically allowed ρ_{ABC} either by distinguishable or indistinguishable particles. For example, take the state described by Paul *et al.* in [3, Supplemental material, Eq. (19)]. This state can only be implemented using indistinguishable particles as shown in [3, Fig. 2]. If in the setup, the particles become distinguishable, then we do not get this state and it does not show a maximum violation of monogamy. Also, it does not show any entanglement at all as shown in [4, Sec. III].

4 Set of three-qubit pure states for distinguishable particles that obeys a strict monogamy relation

In this section, we calculate the list of states that obeys a strict monogamy relation. Let us assume a general state of three-qubit with three particles A, B, and C as

$$|\phi\rangle_{ABC}{=}r_0|000\rangle{+}r_1e^{i\psi}|100\rangle{+}r_2|101\rangle{+}r_3|110\rangle{+}r_4|111\rangle, \tag{4}$$

where $\psi \in [0,\pi]$, $r_i \geq 0$ for $i \in [0,1,2,3,4]$ and $\sum_{i=0}^{4} r^2 = 1$. Now if we calculate the concurrence for ρ_{AB}, ρ_{AC}, and $\rho_{A|BC}$ are

$$\begin{aligned} \mathscr{C}_{A|B}(\rho_{AB}) &= 2r_0r_2, \\ \mathscr{C}_{A|C}(\rho_{AC}) &= 2r_0r_3, \\ \mathscr{C}_{A|BC}(\rho_{A|BC}) &= 2r_0\sqrt{r_2^2+r_3^2+r_4^2}, \end{aligned} \tag{5}$$

where $\rho_{ABC} = |\phi\rangle_{ABC}\langle\phi|_{ABC}$. Now if we calculate the condition for which the state in Eq. (4) will follow a strict monogamy inequality relation is

$$\begin{aligned} \mathscr{C}_{A|BC}^2(\rho_{A|BC}) - \mathscr{C}_{A|B}^2(\rho_{AB}) - \mathscr{C}_{A|C}^2(\rho_{AC}) &> 0 \\ 4r_0^2r_4^2 &> 0. \end{aligned} \tag{6}$$

Thus we have the condition that

$$r_0 \neq 0 \quad \text{and} \quad r_4 \neq 0. \tag{7}$$

So, for the state where we get the condition that Eq. (7) is satisfied, then those states will follow a strict monogamy inequality relation for distinguishable particles. Note that, according to our result in the main paper, these states cannot be produced using indistinguishable particles. Using indistinguishable particles, one can only generate states where $r_0 = 0$ and $r_4 = 0$.

References

1. Yurke, B. & Stoler, D. Bell's-inequality experiments using independent-particle sources. *Phys. Rev. A* **46**, 2229–2234, DOI: 10.1103/PhysRevA.46.2229 (1992).
2. Franco, R. L. & Compagno, G. Quantum entanglement of identical particles by standard information-theoretic notions. *Sci. Reps.* **6**, DOI: 10.1038/srep20603 (2016).
3. Paul, G., Das, S. & Banerji, A. Maximum violation of monogamy of entanglement for indistinguishable particles by measures that are monogamous for distinguishable particles. *Phys. Rev. A* **104**, L010402, DOI: 10.1103/PhysRevA.104.L010402 (2021).
4. Das, S., Paul, G. & Banerji, A. Hyper-hybrid entanglement, indistinguishability, and two-particle entanglement swapping. *Phys. Rev. A* **102**, 052401, DOI: 10.1103/PhysRevA.102.052401 (2020).
